# Infectious Diseases in Children: Diagnosing the Impact of Climate Change-Related Disasters Using Integer-Valued Autoregressive Models with Overdispersion

**DOI:** 10.3390/diseases13090303

**Published:** 2025-09-15

**Authors:** Dessie Wanda, Holivia Almira Jacinta, Arief Rahman Hakim, Atina Ahdika, Suryane Sulistiana Susanti, Khreshna Syuhada

**Affiliations:** 1 Faculty of Nursing, Universitas Indonesia, Depok 16424, Indonesia; dessie@ui.ac.id (D.W.); holivia.almira@ui.ac.id (H.A.J.); 2 Research Centre for Computing, National Research and Innovation Agency (BRIN), Bandung 40135, Indonesia; arief.rahman.hakim@brin.go.id; 3 Department of Statistics, Universitas Islam Indonesia, Yogyakarta 55584, Indonesia; atina.a@uii.ac.id; 4 Statistics Research Division, Institut Teknologi Bandung, Bandung 40132, Indonesia; khreshna@itb.ac.id

**Keywords:** children’s health, climate change, disaster risk, disease incidence, integer-valued time series

## Abstract

The incidence of infectious diseases in children may be affected by climate change-related disaster risks that increase as extreme weather events become more frequent. Therefore, this research aims to diagnose the impact of such disaster risks on the disease incidence, focusing on diarrhoea, dengue haemorrhagic fever (DHF), and acute respiratory infection (ARI), commonly experienced by children. To accomplish this task, we construct integer-valued autoregressive (INAR) models for the number of disease cases among children in several age groups, with an overdispersed distributional assumption to account for its variability that exceeds its central tendency. Additionally, we include the numbers of floods, landslides, and extreme weather events at previous times as explanatory variables. In particular, we consider a case study in Indonesia, a tropical country highly vulnerable to the aforementioned climate change-related diseases and disasters. Using monthly data from January 2010 to December 2024, we find that the incidence of diarrhoea in children is positively impacted by landslides (but negatively affected by floods and extreme weather events). Landslides, frequently caused by excessive rainfall, also increase DHF incidence. Furthermore, the increased incidence of ARI is driven by extreme weather conditions, which are more apparent during and after COVID-19. These findings offer insights into how climate scenarios may increase children’s future health risks. This helps shape health strategies and policy responses, highlighting the urgent need for preventive measures to protect future generations.

## 1. Introduction

Children are particularly vulnerable to infections because their immune systems are still developing, which can lead to severe infectious diseases. This increased vulnerability is also associated with their limited capacity to mitigate the effects of climate change. As climate change drives an increase in the frequency and severity of disasters, infectious diseases become more evident. Post-disaster events due to climate change, such as floods and heatwaves, damage the health infrastructure and worsen sanitation, leading to higher infection rates [1] and, consequently, a higher incidence of infectious diseases, such as diarrhoea, dengue haemorrhagic fever (DHF), and acute respiratory infection (ARI). These three diseases act as the top three causes of death among children in Indonesia for some periods [2,3,4].

Climate-related incidents, such as droughts and extreme temperature fluctuations, increase the risk of respiratory problems in children. When exposed to cold, dry air, or hot air, their bodies may respond with bronchoconstriction and coughing [5]. An increase of 1 °C in temperature can lead to higher respiratory rates, increased incidence of pneumonia, more hospital admissions for pneumonia, and a higher number of emergency department visits due to pneumonia [6]. Furthermore, Chua et al. [7] noted that increased temperatures are associated with an increase in certain infectious diseases caused by bacterial pathogens, such as cholera, salmonella, and *Escherichia coli*. These diseases are associated with both vector-borne and waterborne transmission.

During droughts, people often use unhygienic water and sanitation facilities, leading to gastrointestinal problems, such as diarrhoea, typically the primary health consequence [1]. When children experience diarrhoea, dehydration is the most common health problem they face. Children are also more susceptible to experiencing water loss through evaporation due to severe temperatures since children have a higher total body water content compared to adults [8].

In attempts to conserve water during droughts, individuals often store water in open containers, such as tubs, unintentionally creating ideal breeding environments for mosquitoes and subsequently elevating the risk of DHF. A study by Wibawa et al. [9] suggested that extreme and anomalous weather patterns have a significant impact on the prevalence of DHF, making its seasonal occurrence increasingly unpredictable. In line with this, Thomson et al. [10] also claimed that extreme weather events increase the risk of vector-borne infectious diseases, such as DHF.

Indonesia, as an archipelagic country, is highly vulnerable to climate change, including rising sea levels and tidal flooding [11,12,13]. Climate change in Indonesia appears to worsen with (i) improper waste management practices, (ii) deforestation, (iii) forest fires, (iv) massive exploitation of natural resources without conservation, (v) industrial and vehicle pollution, and (vi) unsustainable agricultural practices [14,15,16,17]. The potential effects on Indonesian children’s health, threatened by infectious diseases, more specifically diarrhoea, DHF, and ARI, are therefore important to investigate.

In this paper, we aim to diagnose the impact of climate change-related disasters (namely, floods, landslides, and extreme weather events) on the incidence of the above infectious diseases (i.e., diarrhoea, DHF, and ARI) in children. It is worth noting that the number of infectious disease cases is a nonnegative integer, leading many studies (e.g., [18,19]) to accommodate its dynamic values over time using an integer-valued time-series model. More specifically, they employed an integer-valued autoregressive (INAR) model introduced independently by McKenzie [20] and Al-Osh and Alzaid [21]. This model was assumed to have a Poisson distribution whose mean and variance are equal, making it equidispersed. In practice, count time-series data on disease cases often exhibit overdispersion, a phenomenon where the variance exceeds the mean [22,23,24]. This phenomenon can be captured using an INAR model with (i) a Poisson–exponential distribution, commonly known as a geometric distribution [22], or (ii) a Poisson–Lindley distribution [25,26]. To the best of our knowledge, these models have not been implemented for medical data on infectious disease cases in children. Furthermore, their extension, which incorporates some exogenous or explanatory variables related to climate change, has not been proposed by the existing studies, thereby inspiring us to construct the so-called INAR-X model with a Poisson–exponential or Poisson–Lindley INAR-X distribution.

We organise the remainder of this paper as follows. In Section 2, we describe the materials and methods, i.e., (i) the sources and visualisation of datasets, which cover the numbers of infectious disease cases among children and climate change-related disaster events, and (ii) INAR models with overdispersion and explanatory variables required to diagnose the impact of the disaster events on the disease incidence. In Section 3, we provide and discuss the results, i.e., statistical analyses that confirm the presence of (i) an overdispersion phenomenon and (ii) a correlation within and between the datasets, along with the main modelling results. We then draw conclusions in Section 4.

## 2. Materials and Methods

### 2.1. Data Sources and Visualisation

#### 2.1.1. Epidemiological Surveillance and Immunisation Information System Database

Data on childhood diseases were obtained from the epidemiological surveillance and immunisation information system database maintained by the Health Office of the Province of the Special Region of Jakarta, Indonesia. Specifically, we focus on three diseases: diarrhoea, dengue haemorrhagic fever (DHF), and acute respiratory infection (ARI). Our focus on Jakarta comes from the fact that this province reflects much of Indonesia through its diverse population and frequent exposure to such diseases in children; see Figure S1 in the Supplementary Materials. This makes our findings more relevant to the whole country. However, we recognise that differences in transmission patterns and population density may affect disease loads in other places, highlighting the necessity of research in provinces that are rural and semi-urban. Furthermore, the selection of the disease variables is based on recent research conducted by Harapan et al. [2], Sulistiyowati et al. [3], and Purnama et al. [4], which revealed that diarrhoea, DHF, and ARI acted as the top three causes of death among children in Indonesia for some periods. See also a study by Helldén et al. [27], which found these to be common diseases faced by children due to climate change.

The above database contains a summary of integrated disease surveillance (IDS). The comprehensive IDS data were compiled from the epidemiological surveillance of both communicable and noncommunicable diseases. These data incorporate information from community health centres, hospitals, laboratories, and the Health Office of Jakarta. In the IDS data, patients’ ages are categorised into twelve distinct groups with nonoverlapping ranges that cover the entire age spectrum, from infancy to old age. The defined age ranges are as follows: 0–7 days, 8–28 days, 29 days–11 months, 12 months–4 years, 5–9 years, 10–14 years, 15–19 years, 20–44 years, 45–54 years, 55–59 years, 60–69 years, and 70 years and older. The first seven are the age groups for children. More detailed IDS data can be accessed, including additional variables such as districts or cities, subdistricts, healthcare centre work units, and sexes.

The primary data source for this study was obtained from the healthcare centres’ IDS database, covering the period from January 2010 to December 2024 and focusing on the Province of the Special Region of Jakarta. It specifically utilises age group information for children of both sexes, within the 0–19 age range. This age range is consistent with the legal definition of a child stated in the Republic of Indonesia Law Number 35 of 2014, which identifies a child as anyone under the age of 18, including those still in the womb [28].

**Figure 1:**
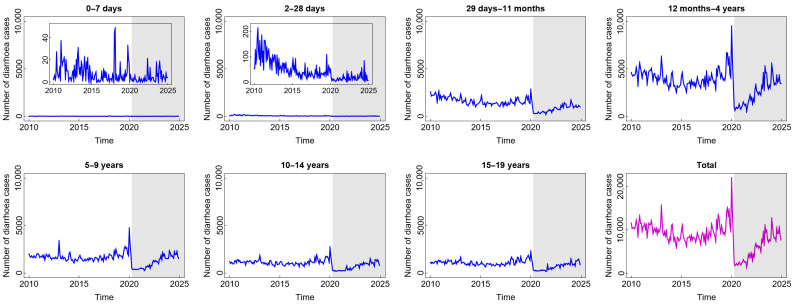
Number of diarrhoea cases among children in several age groups and in the total child population in Jakarta, Indonesia. Notes: The white and grey areas show Period 1 (before the COVID-19 pandemic) and Period 2 (during and after the COVID-19 pandemic), respectively.

The number of diarrhoea cases, as presented in Figure 1, generally follows the same pattern, with the exception of the age group under one month. Before witnessing a notable uptick in 2019 and a precipitous drop in 2020, the number of diarrhoea cases tended to have a stable pattern until around 2018. The year 2020 marked the beginning of the COVID-19 pandemic era, during which most people stayed at home due to widespread social isolation and lockdown-like policies [29]. Accordingly, less data regarding diarrhoea cases were documented. Following that, people were living in a new normal situation, quite similar to their previous life, and the number of diarrhoea cases, particularly for the age groups above one month, tended to increase. The number of cases of this disease did not exhibit the same pattern in the age groups 0–7 days and 8–28 days, indicating that these age groups may have distinct risk factors for transmission compared to the age groups above them. Furthermore, the disease occurrence in these age groups remained unaffected by the circumstances of the COVID-19 pandemic or the new normal. This condition is understandable considering that newborns often receive constant, extremely stringent, and protective care. Only contact with the main family also contributes to limited pathogen contamination, which means that these two age groups likewise experience the fewest cases of diarrhoea. Exclusive breastfeeding and digestive systems that have not been exposed to solid foods can also contribute to a lower incidence in these two age groups. Furthermore, according to Figure 1, the highest number of diarrhoea cases was found in children aged 12 months–4 years. These children are in the stage of exploring their surrounding environment, making them frequently exposed to microorganisms. Due to their hand-to-mouth habits, children under the age of five also have a less developed immune system, which leaves them vulnerable to infections that can cause diarrhoea [30]. In brief, the incidence of diarrhoea in children during and after COVID-19 tended to increase as in the pre-COVID-19 period, even though it dramatically decreased for a short time at the beginning of the COVID-19 outbreak.

**Figure 2:**
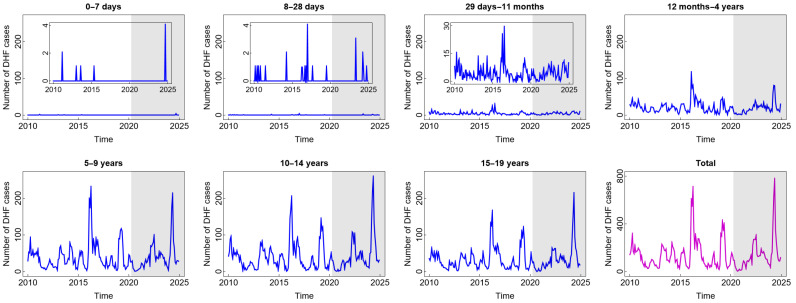
Number of dengue haemorrhagic fever (DHF) cases among children in several age groups and in the total child population in Jakarta, Indonesia. Notes: The white and grey areas show Period 1 (before the COVID-19 pandemic) and Period 2 (during and after the COVID-19 pandemic), respectively.

In general, the pattern of DHF cases was almost the same for all age groups and was not impacted by the COVID-19 pandemic situation (see Figure 2). The high number of DHF cases was found in children aged five years and above. Children in these age groups are more likely to be outside their homes and, therefore, more likely to be exposed to vectors that carry dengue viruses (DENV). A study by Liu et al. [31] confirmed that participation in outdoor activities significantly increases the risk of dengue exposure. Furthermore, research has shown that children under five are not at a high risk of contracting dengue. Younger babies are less likely than older children to have dengue because they may have protective levels of anti-DENV antibodies from their mothers [32]. Interestingly, there was a significant spike in 2016 and 2024, in almost all age groups, with the highest numbers of cases reaching 703 in 2016 and 772 in 2024. A study by Mamenun et al. [33] found a change in serotype from DEN-3 to DEN-1 and DEN-2 in 2016 in Indonesia, which increased the risk of more severe secondary DHF infections. Meanwhile, in 2024 during the dry season, Indonesia experienced a warmer rainy season due to the El Niño phenomenon. A temperature shift and an increase in rainfall during the dry season are anomalies resulting from this occurrence. These anomalies, based on the previous studies, are positively correlated with dengue incidence, where they accelerate the mosquito life cycle and shorten the incubation period of the virus in the mosquito’s body, thus increasing the risk of dengue incidence [34,35].

**Figure 3:**
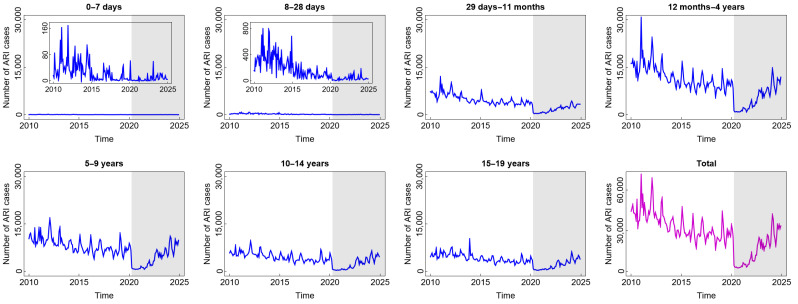
Number of acute respiratory infection (ARI) cases among children in several age groups and in the total child population in Jakarta, Indonesia. Notes: The white and grey areas show Period 1 (before the COVID-19 pandemic) and Period 2 (during and after the COVID-19 pandemic), respectively.

Compared to diarrhoea and DHF, ARI is the most common disease experienced by children in all age groups. From Figure 3, we find that the highest number of total ARI cases in the last 15 years was almost 70,000. The incidence decreased slowly before COVID-19 and then decreased significantly at the beginning of the COVID-19 outbreak. The reason for this phenomenon was similar to the sharp decrease in diarrhoea cases during such a short period. However, the incidence gradually increased from 2022 to 2025 after the new normal phase began to be implemented. Children aged 12 months to 4 years were the group with the highest number of ARI cases. This was also related to the highest mortality rate in children under five due to respiratory infections.

#### 2.1.2. Indonesia Disaster Information Database

The disaster data analysed in this study were derived from the Indonesia Disaster Information Database (DIBI), managed by the National Disaster Management Agency (BNPB) of the Republic of Indonesia. This database provides detailed information about disasters in Indonesia, including their impacts, such as fatalities and economic losses. It organises this information by region, date of occurrence, and types of disaster. The DIBI report encompasses a comprehensive dataset of disasters in Indonesia, spanning from the year 2000, which covers eleven distinct categories of disasters: floods, landslides, wave surges and abrasion, extreme weather, droughts, forest fires, earthquakes, tsunamis, volcanic eruptions, and volcanic explosions. BNPB assigns a unique code to each category of disaster, along with related subsidiary incidents. Users can access DIBI by province and district/city, as well as by year, month, and specific type of disaster.

This research focuses on the Province of the Special Region of Jakarta and considers disasters that occurred in the area from January 2010 to December 2024. In particular, we consider the monthly occurrence of three disasters believed related to climate change: floods, landslides, and extreme weather events; see their recent distributions in Figure S2 in the Supplementary Materials. BNPB classifies various floods under code 101, including coastal floods, flash floods, drainage and trench floods, reservoir floods, stagnant floods, and breaches of levees. Landslides and soil movement are classified as landslide disasters under code 102. Extreme weather events, such as tornadoes, strong windstorms, hurricanes, hailstorms, tropical cyclones, and significant temperature fluctuations, fall under category 104. The Head of BNPB regulates naming conventions and codes for other disaster events in accordance with the Technical Guidelines for Standard Data on Disaster Events and Impacts (Number 7 of 2023) [36].

**Figure 4:**
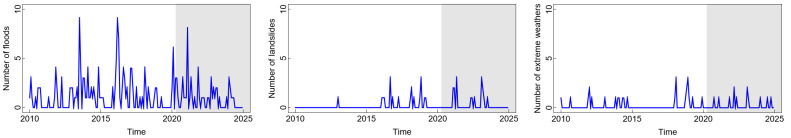
Number of disasters in Jakarta, Indonesia. Notes: The white and grey areas show Period 1 (before the COVID-19 pandemic) and Period 2 (during and after the COVID-19 pandemic), respectively.

In Figure 4, we compare the numbers of floods, landslides, and extreme weather events in Jakarta over the aforementioned period. We observe from the figure that floods occurred more frequently compared to other natural disasters. Floods in Jakarta have become a major issue, not only related to rainfall but also to the management of the water system. The relatively high flood incidents occurred around 2013, 2016, and 2021, with the highest number of cases being nine incidents in both 2013 and 2016. This could be an early indication of its influence on the spike in DHF cases among children in 2016 and the relatively high number of ARI cases in 2013. Meanwhile, although the number of floods in 2021 was also relatively high, the COVID-19 pandemic conditions made this relationship anomalous. On the other hand, the relatively high number of landslides and extreme weather events between 2015 and 2020 can be an early indication of their impact on the number of diarrhoea cases among children approaching 2020. Landslides can worsen sanitation and clean water quality, which can impact the emergence of diseases, such as diarrhoea and cholera [37]. Meanwhile, extreme weather conditions contribute to the possibility of high temperature fluctuations, which accelerate the growth of microorganisms. This is closely related to the increase in cases of waterborne diseases, such as diarrhoea and DHF [38]. It is worth highlighting that although other climate variables, such as rainfall, temperature, and humidity, are recognised as potential factors influencing infectious diseases, they were not included in our analysis and remain important directions for future research.

### 2.2. Integer-Valued Time-Series Models

The widely used model for a time series {*Y_t_*, *t* = 0, 1,…} is an autoregressive (AR) model. Using this model of order one, AR(1), *Y_t_*’s can be expressed as

(1) $$Y_{t}=\beta Y_{t-1}+\varepsilon_{t},$$

where *β* denotes a constant satisfying −1 < *β* < 1, {*ε_t_*, *t* = 0, 1,…} denotes a sequence of independent and identical errors commonly assumed to follow a normal distribution, and *ε_t_* is independent of *Y*_*t*−1_. If we deal with nonnegative *Y_t_*’s, then *β* must satisfy 0 ≤ *β* < 1, and *ε_t_*’s must be nonnegatively distributed according to, for instance, an exponential distribution, as proposed by Gaver and Lewis [39].

In this study, we work with nonnegative integer-valued *Y_t_*’s that specifically represent the numbers of infectious disease cases among children. Hence, we need to replace Equation (1) with the so-called integer-valued autoregressive (INAR) model, as introduced by McKenzie [20] and Al-Osh and Alzaid [21]. This model can express the number of disease cases at a certain time point as the sum of (i) the number of such cases surviving from the previous time point and (ii) the number of new cases at the time point under investigation. The latter term, also taking the value of nonnegative integers, is commonly assumed to have a Poisson distribution whose mean and variance are equal, resulting in a Poisson INAR model that exhibits equidispersion. It was implemented by Cardinal et al. [18] to model the number of meningococcal disease cases reported in the Montréal-Centre region, Canada, and recently by Ahdika and Lusiyana [19] to model the number of DHF patients in West Java, Indonesia. It has also been extended by Brännäs [40] by incorporating some explanatory variables potentially affecting its outcomes. The resulting Poisson INAR-X model was then adopted by Enciso-Mora et al. [41] to model (i) the number of poliomyelitis cases in the United States by involving a time trend or periodicity and (ii) the score achieved by a schizophrenic patient on a test of perceptual speed by including time trend and drug treatment effects. See also similar works by Moriña [42] on modelling the number of hospital emergency service arrivals with influenza symptoms that presented a periodic behaviour; by Syuhada et al. [43] on modelling the health status score of children in Indonesia with consideration of the income of their parents, the education of their mother, and so on; and by Wang [44] on modelling the number of disease cases caused by *Escherichia coli* in North Rhine-Westphalia, Germany, with the inclusion of a time trend.

In practice, count time-series data on disease cases frequently show overdispersion, a phenomenon where the variance exceeds the mean. This evidence was highlighted by Maiti and Biswas [22] when analysing the numbers of poliomyelitis cases in the United States (see also [23]) and submissions with skin lesions from a region in New Zealand to animal health laboratories and by Mojica and Co [24] when detecting measles cases during the largest measles outbreak in the Philippines. The overdispersion in these data cannot be captured by the classical Poisson INAR model, thereby motivating the use of a geometric INAR model as an alternative. They demonstrated that using a geometric distributional assumption resulted in an overdispersed INAR model with superior performance compared to the Poisson INAR model. Basically, a geometric distribution can be viewed as a mixed Poisson distribution, namely, a Poisson–exponential distribution, where the Poisson parameter is assumed to follow an exponential distribution. Another important one is Sankaran’s [45] Poisson–Lindley distribution, which is also overdispersed. Its use to construct an INAR model has been taken into consideration by Lívio et al. [25] and Mohammadpour et al. [26]. (It has also been utilised to construct a continuous-time stochastic model by Cha [46] and Syuhada et al. [47]). In particular, Mohammadpour et al. [26] implemented the resulting Poisson–Lindley INAR model for count time-series data on submissions with skin lesions and anorexia from a region in New Zealand to animal health laboratories.

#### 2.2.1. INAR Models

An INAR model of order one, INAR(1), is formulated as follows [20,21,48]:

(2) $$Y_{t}=\beta \circ Y_{t-1}+\varepsilon_{t},$$

where $\beta \circ Y_{t-1}=B_{1}+B_{2}+\ldots +B_{Y_{t-1}}$ for some sequence {*B_i_*, *i* = 1, 2,…} of independent and identical binary random variables with probability distribution $P(B_{i}=1)=\beta$ and $P(B_{i}=0)=1-\beta$, 0≤β<1. This means that at time t−1, there exist $Y_{t-1}$ different children diagnosed with a certain disease. At time *t*, each of them may still have the disease with probability β or may not have the disease with probability 1−β. Thus, at time *t*, there are $\beta \circ Y_{t-1}$ children who have this disease along with additional $\varepsilon_{t}$ children diagnosed with the same disease. Their sum, $Y_{t}$, given in Equation (2), has a mean, a variance, and a dispersion index written, respectively, as follows [49,50]:

(3) $$E\left(Y_{t}\right)=\frac{E(\varepsilon_{t})}{1-\beta },\;V\left(Y_{t}\right)=\frac{V\left(\varepsilon_{t}\right)+\beta E\left(\varepsilon_{t}\right)}{1-\beta^{2}},\;I\left(Y_{t}\right)=\frac{V(Y_{t})}{E(Y_{t})}=\frac{I(\varepsilon_{t})+\beta }{1+\beta },$$

where $E(\varepsilon_{t})$, $V(\varepsilon_{t})$, and $I\left(\varepsilon_{t}\right)=V(\varepsilon_{t})/E\left(\varepsilon_{t}\right)$, respectively, denote the mean, variance, and dispersion index of $\varepsilon_{t}$. This indicates that $I(\varepsilon_{t})=1$ implies $I(Y_{t})=1$ (equidispersion), and $I(\varepsilon_{t})>1$ implies $I(Y_{t})>1$ (overdispersion).

#### 2.2.2. INAR Models with Overdispersion

One commonly assumes that the error term $\varepsilon_{t}$ follows a Poisson distribution with parameter λ>0, which is equal to its mean and variance. Consequently, the resulting Poisson INAR(1) model for $Y_{t}$ is equidispersed. In this study, we consider $\varepsilon_{t}$ to have a mixed Poisson distribution by assuming that the Poisson parameter λ is a value of a nonnegative random variable Λ. In particular, if Λ follows an exponential distribution with parameter γ>0, then $\varepsilon_{t}$ possesses a Poisson–exponential distribution, well known as a geometric distribution. Under this distributional assumption, the resulting Poisson–exponential INAR(1) model is overdispersed. Alternatively, if Λ obeys a Lindley distribution with parameter γ [51], then $\varepsilon_{t}$ has a Poisson–Lindley distribution [45]. In this case, we obtain a Poisson–Lindley INAR(1) model that is also overdispersed. See Table 1 for more details.

#### 2.2.3. INAR Models with Explanatory Variables

We now assume that there are some explanatory variables *X*_1,*t*−1_, *X*_2,*t*−1_,…, *X*_*m*,*t*−1_ that specifically denote the numbers of *m* different disasters at the previous time *t* − 1. To incorporate them, we adopt an approach by Brännäs [40] and Enciso-Mora et al. [41] to extend the classical INAR(1) model to become an INAR(1)-X model. We can express the latter model as

(4) $$Y_{t}=\beta_{t}\circ Y_{t-1}+\varepsilon_{t}$$

with a time-varying probability parameter

(5) $$\beta_{t}=\frac{1}{1+\exp(-\left(b_{0}+b_{1}X_{1,t-1}+\ldots +b_{m}X_{m,t-1}\right))}$$

for some constants *b*_0_, *b*_1_,…, *b_m_*. In particular, we propose Poisson–exponential INAR(1)-X and Poisson–Lindley INAR(1)-X models, each of which has a time-varying parameter

(6) $$\gamma_{t}=\exp\left(-\left(c_{0}+c_{1}X_{1,t-1}+\ldots +c_{m}X_{m,t-1}\right)\right)$$

for some constants *c*_0_, *c*_1_,…, *c_m_*. It is worth mentioning that in their original works, Brännäs [40] and Enciso-Mora et al. [41] considered a time-varying probability parameter of the form $\beta_{t}=1/(1+\exp\left(b_{0}+b_{1}X_{1t}+\ldots +b_{m}X_{mt}\right))$, which depends on the explanatory variables at time *t* and is a decreasing function of *b_j_*, *j* = 0, 1,…, *m*; they also assumed that the error term *ε_t_* has a Poisson distribution with time-varying parameter $\lambda_{t}=\exp\left(c_{0}+c_{1}X_{1t}+\ldots +c_{m}X_{mt}\right)$, which is an increasing function of *b*_j_, *j* = 0, 1,…, *m*. Meanwhile, our proposed logistic link function for $\beta_{t}$ in Equation (5) is set to ensure that it depends on the explanatory variables at the previous time *t* − 1 and is increasing with respect to *b_j_*, *j* = 0, 1,…, *m*. Similarly, Equation (6) results in a time-varying $E(\varepsilon_{t})$ that is increasing with respect to *c_j_*, *j* = 0, 1,…, *m*. As a consequence, the time-varying conditional mean of $Y_{t}$, i.e., $E\left(Y_{t}|Y_{t-1}\right)=\beta_{t}\;Y_{t-1}+E\left(\varepsilon_{t}\right)$, is also increasing with respect to both *b_j_* and *c_j_*, *j* = 0, 1,…, *m*.

## 3. Results and Discussion

### 3.1. Descriptive Statistics

In Table 2, we provide a summary of the statistics for infectious disease cases among children, including the mean, variance, skewness, kurtosis, and dispersion index. According to the mean value, the most cases of diarrhoea and ARI occurred in children aged 1–4 years, whereas the majority of DHF cases occurred in the 5–14-year age group. Similarly, the highest variance value was seen in the same age groups for each kind of disease. This demonstrates that, in addition to having the largest number of cases, the disease occurrences vary significantly in such age groups. This suggests that in these age groups, changes in environmental and behavioural factors are more susceptible to effect. While most cases of diarrhoea and ARI occurred in children aged 1–4 years, they decreased almost by half over the period during and after the COVID-19 pandemic. A different pattern was found in DHF cases, where it increased in several age groups due to the pandemic. Meanwhile, a dispersion index value larger than one (which is in line with a positive coefficient of skewness) in all disease categories and age groups indicates that the data are overdispersed, with extremely large dispersion in the diarrhoea and ARI case datasets. This evidence tends to be more pronounced during and after the pandemic, making a Poisson distribution less applicable. Floods were the most common disaster, followed by landslides and extreme weather events (see Table 3). When compared to the other two disasters, floods also had the comparatively highest case variability and dispersion.

### 3.2. Correlation Analysis

In Figures S3–S6 in the Supplementary Materials, we depict the autocorrelation and partial autocorrelation functions for diarrhoea, DHF, and ARI cases as well as disaster events, respectively. For diarrhoea and ARI, there was evidence of strongly positive autocorrelations between the number of cases in a certain month and those in previous months for all age groups, with the strongest autocorrelation occurring at a one-month lag. The autocorrelations for age groups beyond 28 days became stronger as the COVID-19 pandemic progressed. Meanwhile, in contrast to the other two types of disease, the numbers of DHF cases were weakly positively autocorrelated at large lags, with the weakest autocorrelations occurring in children aged below one month. Due to evidence from Figure 2 that DHF cases were not impacted by the COVID-19 pandemic, their autocorrelations tended to remain unchanged. For all disease types, the significance of their partial autocorrelation only at a one-month lag led us to consider an integer-valued autoregressive model of order one, INAR(1). For disaster events, despite being unclearly apparent, their autocorrelation at a one-month lag is sufficient to provide an argument that each disaster event in a certain month *t* was influenced by the same event in the previous month *t* − 1.

Furthermore, in Figures S7–S9 in the Supplementary Materials, we provide the cross-correlation function between the number of infectious disease cases in children in month *t* and the number of natural disasters in the previous month *t* − 1. We observe from the figures that flood and landslide occurrences tended to be positively cross-correlated with DHF cases, although the cross-correlation became weaker following the COVID-19 outbreak. In contrast, they were negatively cross-correlated with diarrhoea and ARI cases. Meanwhile, extreme weather events tended to have positive but weak cross-correlations with all disease cases. The same patterns appeared even when the numbers of disaster events were transformed using an exponential or logistic function. This suggests the presence of nonlinear relationships between disease cases and disaster occurrences, thereby allowing us to incorporate the latter as explanatory variables nonlinearly impacting the former in our proposed INAR(1)-X model.

### 3.3. Time-Series Modelling

Using the available data described in previous subsections, we estimate the parameters *b*_0_, *b*_1_, *b*_2_, *b*_3_, *c*_0_, *c*_1_, *c*_2_, *c*_3_ of the Poisson–exponential INAR(1)-X and Poisson–Lindley INAR(1)-X models for the numbers of cases of each infectious disease in children. We accomplish this task by involving the numbers of the three disasters—floods, landslides, and extreme weather events—as explanatory variables. We provide the parameter estimation results in Table 4, Table 5 and Table 6. Through a comparative analysis with the Nash–Sutcliffe efficiency (NSE), we reveal that the two models have similar accuracy and interchangeably perform best. Furthermore, as shown in Figures S10–S15 in the Supplementary Materials, the resulting errors or residuals have no (partial) autocorrelation, indicating that they are randomly and independently distributed.

According to Table 4, regardless of the effects of natural disasters, ${\hat{b}}_{0}$ values, which are positive for almost all age groups, indicate that children diagnosed with diarrhoea tend to remain diagnosed with the same disease in the following month with probability $\hat{\beta }=1/[1+\exp\left(-{\hat{b}}_{0}\right)]$ greater than 0.5. When examining the relationships between diarrhoea incidences and disaster occurrences affecting children, the statistical analysis indicates that disaster events have a relatively significant impact on the probability that a child will remain diagnosed with diarrhoea (see the $\hat{b}$ columns) and on the additional new children diagnosed with diarrhoea in the following month (see the $\hat{c}$ columns). However, floods (and extreme weather events) tend to have relatively negative effects. This finding is contrary to other studies, which demonstrated that flooding is associated with increased diarrhoea cases due to a lack of access to clean water [54] and a higher risk of waterborne vector transmission [55]. Moreover, Wang et al. [56] noted that a heightened risk of diarrhoea can occur during severe flooding events that last for extended periods. This scenario does not align with conditions in Indonesia, where nonextreme floods occur ten times more frequently than flash floods. Furthermore, the average duration of flood inundation is approximately three days, which starkly contrasts with flood events that occur on a weekly or monthly basis [57,58].

Table 5 unequivocally shows that even without the influence of disasters, children remain at a significant risk of contracting dengue haemorrhagic fever (DHF). The incidence of DHF can be influenced by several environmental factors, which tend to play a more dominant role. These factors include geographical variation, climate, mosquito breeding sites, and preventive measures, such as mosquito control practice [59]. Statistical evidence clearly establishes a significantly positive relationship between floods/landslides and the increased risk of DHF. While landslides do not directly lead to DHF, they are frequently triggered by excessive rainfall [60]. Excessive rainfall with inadequate water absorption often creates puddles that serve as an ideal breeding site for mosquitoes [61]. Additionally, high rainfall increases air humidity, a key factor that facilitates the breeding of *Aedes aegypti* mosquitoes [62]. Additionally, the forced relocation of residents affected by landslides to other areas significantly escalates the risk of DHF, as it leads to increased population density and the establishment of new mosquito habitats.

The statistical modelling results in Table 6 clearly demonstrate that, even in the absence of disaster variables, a baseline risk of acute respiratory infections (ARIs) remains significant and is likely to increase. These results also show the statistical significance of a strongly positive relationship between extreme weather conditions and the likelihood of ARI cases in children, particularly during and after COVID-19. This heightened risk can be directly attributed to their weak respiratory systems that increase susceptibility to infections [63]. Therefore, immunisation programs become extremely important to be applied in Indonesia. Vaccines can save lives by imitating an infection triggered by weakened or killed microorganisms [64,65].

Furthermore, maternal demographic factors significantly affect the risk of ARI, as young mothers (under 20 years of age) are 2.02 times more likely to give birth to babies with low birth weight (LBW). Agho et al. [66] and Ejigu et al. [67] mentioned that young mothers tend not to feed their infants and often give their infants formula milk due to a lack of experience, education, attitudes, and social support. Nevertheless, given that breast milk contains vital natural antibodies crucial for combating infections and enhancing immune responses, inadequate breastfeeding further exacerbates the risk [68]. Moreover, socioeconomic conditions and geographic factors undeniably contribute to the prevalence of ARI. Families with low socioeconomic status typically live in poorly ventilated and densely populated environments, which worsen health outcomes [69].

In comparing our time-series model with those reported in previous studies, we note that, unlike many models that rely primarily on climate variables, our approach uses disaster events to diagnose disease outcomes, offering practical value in disaster-prone settings. Although validation and local adaptation are still required, this highlights the model’s contribution to improving diagnostic tools for climate-related health risks.

The utilisation of the time-series model’s framework for data from Jakarta is adaptable and could be applied in other regions with comparable disaster and disease surveillance data. Its use elsewhere will require validation and adjustment to local epidemiological, demographic, environmental, and health system conditions and may include spatial or spatiotemporal effects among different regions, as in recent studies by, e.g., Alene et al. [70] and Silveira et al. [71]. Such a work would confirm its transferability and strengthen its value for global climate-related health risk assessment.

## 4. Conclusions

This study highlights the urgent need for diagnostic tools to protect children in Indonesia from the health impacts of climate-related disasters. The results demonstrate that landslides (floods and extreme weather events) tend to increase (decrease) the incidence of diarrhoea in children. Floods and landslides, linked to heavy rainfall, are also expected to drive dengue haemorrhagic fever (DHF), whereas extreme weather events sharply elevate acute respiratory infection (ARI), particularly during and after the COVID-19 pandemic.

Our findings suggest that disaster events such as floods, landslides, and extreme weather events are often followed by short-term rises in certain infectious diseases such as diarrhoea, DHF, and ARI in children. While these increases may appear temporary, they add to the overall disease burden, place additional pressure on health services, and, in settings with high vulnerability or limited capacity, can escalate into severe public health emergencies. Recognising these patterns provides an important evidence base for strengthening early warning systems, improving the allocation of health resources, and shaping disaster risk reduction and public health strategies in Indonesia.

While this study centres on Jakarta, the city’s diverse population, frequent exposure to infectious diseases in children, and frequent encounters with climate-related disasters make it useful as a reflection for wider Indonesia or even more broadly to other cities in the world that have similar conditions. We acknowledge that patterns of disease transmission, differences in population density, and the varied impacts of disasters may shape health outcomes differently in other settings. This underscores the need for further research in rural and semi-urban provinces, where the risks of disasters differ and where there is an even greater urgency to design interventions that protect the health of children.

## Figures and Tables

**Table 1:** Distributional assumptions for $\varepsilon_{t}$ in the INAR(1) model for $Y_{t}$.

Distribution for $\varepsilon_{t}$	$P(\varepsilon_{t}=\ell )$	$E(\varepsilon_{t})$	$V(\varepsilon_{t})$	$I(\varepsilon_{t})$	$I(Y_{t})$
Poisson–exponential (γ)	$\frac{\gamma }{\gamma +1}{\left(\frac{1}{\gamma +1}\right)}^{\ell }$	$\frac{1}{\gamma }$	$\frac{\gamma +1}{\gamma^{2}}$	$1+\frac{1}{\gamma }>1$	>1 (overdispersion)
Poisson–Lindley (γ)	$\frac{\gamma^{2}\left(\ell +\gamma +2\right)}{{\left(\gamma +1\right)}^{\ell +3}}$	$\frac{\gamma +2}{\gamma (\gamma +1)}$	$\frac{\gamma^{3}+4\gamma^{2}+6\gamma +2}{\gamma^{2}{\left(\gamma +1\right)}^{2}}$	$1+\frac{\gamma^{2}+4\gamma +2}{\gamma (\gamma +1)(\gamma +2)}>1$	>1 (overdispersion)

Notes: See Livio et al. [25], Jazi et al. [52], and Ghitany and Al-Mutairi [53] for further details.

**Table 2:** Descriptive statistics of count time-series data on infectious disease cases among children over Period 1 (before the COVID-19 pandemic) and Period 2 (during and after the COVID-19 pandemic).

Age Group	Statistic	Diarrhoea		DHF		ARI	
		Period 1	Period 2	Period 1	Period 2	Period 1	Period 2
0–7 days	Mean	8.34	3.96	0.04	0.07	28.00	6.21
	Variance	79.83	21.18	0.06	0.28	988.16	84.95
	Skewness	1.96	1.92	6.36	7.35	1.83	2.97
	Kurtosis	7.66	6.27	46.01	55.02	7.28	14.97
	Dispersion	**9.57**	**5.34**	**1.37**	**4.00**	**35.29**	**13.68**
8–28 days	Mean	59.88	15.68	0.14	0.12	215.83	33.81
	Variance	1670.32	178.18	0.23	0.25	28,360.11	1084.91
	Skewness	1.36	2.43	5.12	4.50	1.31	2.21
	Kurtosis	4.61	10.77	36.04	23.42	4.63	8.16
	Dispersion	**27.90**	**11.36**	**1.70**	**2.06**	**131.40**	**32.09**
29 days–11 months	Mean	1635.46	775.91	4.41	4.21	4898.54	1748.68
	Variance	160,695.69	127,418.83	19.36	9.31	2,666,240.20	1,167,432.22
	Skewness	0.62	0.30	2.61	0.51	1.30	0.33
	Kurtosis	2.60	2.34	13.08	2.27	5.05	2.08
	Dispersion	**98.26**	**164.22**	**4.39**	**2.21**	**544.29**	**667.61**
12 months–4 years	Mean	4080.89	2505.16	21.72	18.68	11,945.51	5255.67
	Variance	900,079.34	1753,768.24	263.99	271.43	14,430,398.60	14,022,945.23
	Skewness	1.88	0.32	2.33	2.07	1.47	0.49
	Kurtosis	10.25	2.09	12.08	8.26	6.91	2.19
	Dispersion	**220.56**	**700.06**	**12.15**	**14.53**	**1208.02**	**2668.16**
5–9 years	Mean	1717.45	1134.40	39.88	38.47	8229.88	4151.86
	Variance	199,010.33	404,042.39	1383.04	1470.08	5,529,578.22	9,608,725.41
	Skewness	3.01	0.20	2.35	2.53	0.88	0.51
	Kurtosis	17.83	1.70	10.56	10.91	3.91	2.12
	Dispersion	**115.88**	**356.17**	**34.68**	**38.21**	**671.89**	**2314.32**
10–14 years	Mean	1125.36	678.60	39.20	44.26	4633.88	2317.72
	Variance	76,707.17	150,724.57	1285.97	2402.34	1,892,112.73	3,182,916.42
	Skewness	1.83	0.28	1.79	2.48	0.70	0.50
	Kurtosis	9.76	1.88	7.18	9.90	3.47	1.96
	Dispersion	**68.16**	**222.11**	**32.80**	**54.27**	**408.32**	**1373.30**
15–19 years	Mean	1095.85	670.79	32.66	34.19	4207.86	2011.28
	Variance	72,759.12	135,860.78	836.49	1318.52	1,773,481.51	2,288,682.99
	Skewness	1.17	0.33	1.90	2.85	1.02	0.68
	Kurtosis	5.06	2.14	7.39	13.02	4.91	2.47
	Dispersion	**66.40**	**202.54**	**25.61**	**38.56**	**421.47**	**1137.92**
Total	Mean	9723.23	5784.51	138.06	140.02	34,159.50	15,525.23
	Variance	4,719,290.80	9,066,456.29	13,843.25	19,533.09	102,428,067.87	124,122,128.07
	Skewness	1.74	0.21	2.06	2.60	0.98	0.48
	Kurtosis	9.57	1.88	8.79	11.09	4.23	2.11
	Dispersion	**485.36**	**1567.37**	**100.27**	**139.50**	**2998.52**	**7994.87**

Notes: The dispersion index of each dataset is a ratio between its variance and mean. The dispersion index given in boldface is greater than one, indicating overdispersion.

**Table 3:** Descriptive statistics of count time-series data on natural disasters over Period 1 (before the COVID-19 pandemic) and Period 2 (during and after the COVID-19 pandemic).

Statistic	Flood		Landslide		Extreme Weather	
	Period 1	Period 2	Period 1	Period 2	Period 1	Period 2
Mean	1.21	1.04	0.15	0.30	0.21	0.21
Variance	3.05	1.96	0.25	0.53	0.30	0.24
Skewness	2.17	2.53	3.94	2.52	3.10	2.28
Kurtosis	8.76	12.03	20.19	8.48	13.64	7.44
Dispersion	**2.52**	**1.90**	**1.60**	**1.79**	**1.42**	**1.14**

Notes: The dispersion index of each dataset is a ratio between its variance and mean. The dispersion index given in boldface is greater than one, indicating overdispersion.

**Table 4:** Estimated parameters and Nash–Sutcliffe efficiency of INAR(1)-X models for the number of diarrhoea cases among children over Period 1 (before the COVID-19 pandemic) and Period 2 (during and after the COVID-19 pandemic).

Age Group	Model	${\hat{b}}_{0}$	${\hat{b}}_{1}$	${\hat{b}}_{2}$	${\hat{b}}_{3}$	${\hat{c}}_{0}$	${\hat{c}}_{1}$	${\hat{c}}_{2}$	${\hat{c}}_{3}$	NSE
Period 1										
0–7 days	PE-INAR(1)-X	−0.33	−0.60 *	−7.11 *	2.12 *	1.77 *	−0.01	−0.05	−0.40	**26.44%**
	PL-INAR(1)-X	−0.33	−0.60 *	−7.08 *	2.12*	1.20 *	−0.01	−0.04	−0.36	26.43%
8–28 days	PE-INAR(1)-X	0.22	0.34 *	−0.55 *	0.56	3.45 *	−0.27 *	−0.64 *	−0.88 *	**43.04%**
	PL-INAR(1)-X	0.22	0.34 *	−0.54 *	0.57	2.78 *	−0.25 *	−0.62 *	−0.86 *	43.03%
29 days–11 months	PE-INAR(1)-X	0.43 *	−0.04	0.21 *	−0.18 *	6.53 *	−0.03	−0.27 *	0.10 *	**42.72%**
	PL-INAR(1)-X	0.43 *	−0.04	0.21 *	−0.18 *	5.84 *	−0.03	−0.27 *	0.10 *	42.72%
12 months–4 years	PE-INAR(1)-X	2.91 *	−0.20 *	0.96	884.39	5.65 *	−86.20 *	−104.83 *	0.10	11.58%
	PL-INAR(1)-X	0.30 *	−0.19 *	−0.24 *	0.19	6.83 *	0.04 *	0.04 *	−0.03	**32.65%**
5–9 years	PE-INAR(1)-X	0.42 *	−0.24 *	−0.54 *	1.05 *	6.59 *	0.07 *	0.14 *	−0.51 *	**31.58%**
	PL-INAR(1)-X	0.37 *	−0.19 *	−0.61 *	6.40	5.91 *	0.06 *	0.16 *	−22.58 *	29.77%
10–14 years	PE-INAR(1)-X	0.24 *	−0.21 *	0.02	−0.44 *	6.27 *	0.05 *	−0.11 *	0.22 *	28.96%
	PL-INAR(1)-X	0.24 *	−0.21 *	0.02	−0.44 *	5.58 *	0.05 *	−0.11 *	0.22 *	**28.96%**
15–19 years	PE-INAR(1)-X	0.49 *	−0.27 *	0.31 *	−0.44 *	6.10 *	0.07 *	−0.27 *	0.32 *	**41.41%**
	PL-INAR(1)-X	0.49 *	−0.27 *	0.31 *	−0.44 *	5.41 *	0.07 *	−0.27 *	0.32 *	41.41%
Total	PE-INAR(1)-X	0.38 *	−0.19 *	−0.46 *	0.09	8.35 *	0.04 *	0.11 *	0.03	34.16%
	PL-INAR(1)-X	0.38 *	−0.19 *	−0.46 *	0.09	7.66 *	0.04 *	0.11 *	0.03	**34.16%**
Period 2										
0–7 days	PE-INAR(1)-X	−87.35 *	87.72 *	85.12 *	1.16 *	1.63 *	−0.77 *	−0.42	−0.60 *	48.94%
	PL-INAR(1)-X	−53.89 *	54.13 *	19.14 *	−109.25 *	1.03 *	−0.57 *	−0.06	−0.10	**51.37%**
8–28 days	PE-INAR(1)-X	−25.44 *	−4.63	−4.96	−5.68 *	2.74 *	0.04	−0.11 *	0.02	**58.20%**
	PL-INAR(1)-X	−22.34 *	−20.09 *	−9.23	−7.14 *	2.10 *	0.04	−0.10 *	0.01	58.20%
29 days–11 months	PE-INAR(1)-X	68.09 *	−10.22 *	12.17 *	9.42 *	4.27 *	−15.80	−1.17	−112.49 *	94.83%
	PL-INAR(1)-X	4.86 *	−0.54 *	9.55 *	−1.55 *	−526.71 *	27.13 *	149.54 *	14.45 *	**95.22%**
12 months–4 years	PE-INAR(1)-X	4.44 *	−0.36 *	31.45 *	−1.63 *	−34.32 *	−15.45 *	3.38 *	−13.51 *	93.98%
	PL-INAR(1)-X	1.84 *	−0.11	0.22	0.27	5.72 *	−0.37 *	−0.45	−2600.49	**94.62%**
5–9 years	PE-INAR(1)-X	2.54 *	0.04	16.82	−0.71 *	5.24 *	−0.57	−1.70	−18.39 *	**96.05%**
	PL-INAR(1)-X	4.07 *	0.12	32.73 *	−1.84 *	−33.08 *	−32.66 *	2.07 *	−18.07 *	95.61%
10–14 years	PE-INAR(1)-X	1.56 *	−0.20 *	213.19 *	0.35 *	5.12 *	−0.12	−1.08	−1.23	92.88%
	PL-INAR(1)-X	1.55 *	−0.19 *	1.38	0.30	4.45 *	−0.15	−0.65	−1.06	**92.88%**
15–19 years	PE-INAR(1)-X	3.91 *	−0.44 *	31.80 *	−1.19 *	−38.11 *	−9.63 *	5.10 *	1.79 *	91.82%
	PL-INAR(1)-X	1.81 *	−0.39 *	0.31 *	−0.18	4.19 *	0.10 *	−0.15	−0.12	**92.64%**
Total	PE-INAR(1)-X	2505.99 *	−0.02	987.54	−2504.27 *	-28.40 *	2.46 *	−0.99 *	17.09 *	95.48%
	PL-INAR(1)-X	2.17 *	−0.15	0.61	-0.10	6.32 *	−0.34	−0.64	−17.93 *	**95.57%**

Notes: PE and PL, respectively, stand for the Poisson–exponential and Poisson–Lindley error distributions for the INAR(1)-X model $Y_{t}=\beta_{t}\circ Y_{t-1}+\varepsilon_{t}$, as characterised in Table 1. Meanwhile, NSE is the Nash–Sutcliffe efficiency, as formulated in Equation (A6) in Appendix A; the highest NSE is given in boldface. The parameters $b_{0},b_{1},b_{2},b_{3}$ determine the probability $\beta_{t}=1/[1+\exp\left(-\left(b_{0}+b_{1}X_{1,t-1}+b_{2}X_{2,t-1}+b_{3}X_{3,t-1}\right)\right)]$ that a child will still have diarrhoea from month t−1 to month *t*, where $X_{1,t-1},X_{2,t-1},X_{3,t-1}$, respectively, denote the numbers of floods, landslides, and extreme weather events in the previous month t−1. Meanwhile, $c_{0},c_{1},c_{2},c_{3}$ determine the parameter $\gamma_{t}=\exp\left(-\left(c_{0}+c_{1}X_{1,t-1}+c_{2}X_{2,t-1}+c_{3}X_{3,t-1}\right)\right)$ of the error or additional number $\varepsilon_{t}$ of diarrhoea cases from month t−1 to month *t*. Positive (negative) $b_{j}$ and $c_{j}$ indicate that an increase in $X_{j,t-1}$ results in an increase (decrease) in the probability $\beta_{t}$ and the mean $E(\varepsilon_{t})$. The asterisk * suggests that $b_{j}$ and $c_{j}$ are significantly nonzero at the 5% significance level, implying that there is a significant effect of $X_{j,t-1}$ on $\beta_{t}$ and $E(\varepsilon_{t})$, and also on $E(Y_{t})$.

**Table 5:** Estimated parameters and Nash–Sutcliffe efficiency of INAR(1)-X models for the number of DHF cases among children over Period 1 (before the COVID-19 pandemic) and Period 2 (during and after the COVID-19 pandemic).

Age Group	Model	${\hat{b}}_{0}$	${\hat{b}}_{1}$	${\hat{b}}_{2}$	${\hat{b}}_{3}$	${\hat{c}}_{0}$	${\hat{c}}_{1}$	${\hat{c}}_{2}$	${\hat{c}}_{3}$	NSE
Period 1										
0–7 days	PE-INAR(1)-X	−38.70 *	0.74 *	9.81 *	−6.74	−2.81 *	−23.43 *	−17.88 *	−24.21 *	−0.37%
	PL-INAR(1)-X	−37.18 *	3.62 *	9.33 *	0.14	−2.87 *	−17.27 *	−20.78 *	−20.21 *	**–0.37%**
8–28 days	PE-INAR(1)-X	−70.04 *	−5.46 *	−3.31 *	−0.99	−2.03 *	0.14 *	0.05	−11.78 *	**2.56%**
	PL-INAR(1)-X	−40.92 *	10.35 *	−3.51 *	−0.58	−1.84 *	−33.45 *	−28.14 *	−24.40 *	0.88%
29 days–11 months	PE-INAR(1)-X	0.09	−0.62 *	−0.17	−1.08	0.69 *	0.20 *	0.23 *	−0.10	**19.17%**
	PL-INAR(1)-X	0.10	−0.63 *	−0.16	−1.09	0.25 *	0.18 *	0.20 *	−0.09	18.94%
12 months–4 years	PE-INAR(1)-X	0.57 *	0.13 *	−0.32	−0.54	1.88 *	0.07	−1.36 *	0.52 *	**45.99%**
	PL-INAR(1)-X	0.58 *	0.13 *	−0.32	−0.56	1.29 *	0.06	−1.31 *	0.50 *	45.97%
5–9 years	PE-INAR(1)-X	0.08	1.44 *	1.62 *	1.95	2.36 *	−0.59 *	0.97 *	−0.87 *	58.98%
	PL-INAR(1)-X	0.41 *	0.86	18.84 *	−0.31	1.45 *	−0.07	−1.85 *	0.78 *	**61.64%**
10–14 years	PE-INAR(1)-X	0.32 *	2.79 *	−5.11 *	4.72 *	2.08 *	−1.98 *	2.76 *	−1.91 *	**67.87%**
	PL-INAR(1)-X	0.81 *	1.83	31.76	2.09	0.31 *	0.01	−2.09 *	1.09 *	67.45%
15–19 years	PE-INAR(1)-X	0.43 *	40.97 *	−21.47 *	1.21	1.81 *	−25.66 *	2.81 *	−0.88	62.42%
	PL-INAR(1)-X	0.76 *	2.05 *	18.61 *	−0.20	0.58 *	0.08	−1.96 *	0.90 *	**65.10%**
Total	PE-INAR(1)-X	0.89 *	1.54	17.98	0.35	2.44 *	0.03	−2.25 *	1.00 *	**66.38%**
	PL-INAR(1)-X	0.44 *	2.60 *	−4.55 *	3.27	2.68 *	−1.82 *	2.57 *	−1.77 *	64.34%
Period 2										
0–7 days	PE-INAR(1)-X	−27.10 *	0.14	0.15	0.16	−1.39	−8.84 *	−8.35 *	−8.41 *	**4.55%**
	PL-INAR(1)-X	−27.25 *	0.14	0.15	0.16	−1.55 *	−7.99 *	−7.48 *	−7.55 *	4.55%
8–28 days	PE-INAR(1)-X	−7.48 *	5.34 *	0.00	−2.46 *	−1.67 *	−0.76	0.54	−12.90 *	**5.32%**
	PL-INAR(1)-X	−7.85 *	5.70 *	−0.21	−11.81 *	−1.80 *	−0.68	0.47	−16.81 *	5.28%
29 days–11 months	PE-INAR(1)-X	−18.69 *	19.40 *	19.83 *	37.99 *	1.16 *	−0.60 *	−0.70	−0.94	**39.14%**
	PL-INAR(1)-X	−16.12 *	16.82 *	17.23 *	101.74 *	0.66 *	−0.53 *	−0.60	−0.84	39.12%
12 months–4 years	PE-INAR(1)-X	−1.21 *	2.33 *	1.20 *	1.06	2.33 *	−0.65	0.09	0.01	**59.40%**
	PL-INAR(1)-X	−1.19 *	2.30 *	1.19 *	1.06	1.71 *	−0.58	0.09	0.01	59.39%
5–9 years	PE-INAR(1)-X	−0.40	1.51	23.24 *	−0.27	2.66 *	−0.20	−19.38 *	0.21	60.91%
	PL-INAR(1)-X	−0.40	1.51	34.71 *	−0.27	2.03 *	−0.19	−30.56 *	0.20	**60.91%**
10–14 years	PE-INAR(1)-X	0.19	1.56	2.14 *	−0.81 *	2.29 *	−0.26 *	−18.76 *	1.11 *	**70.50%**
	PL-INAR(1)-X	0.18	1.57	2.14 *	−0.78 *	1.68 *	−0.25 *	−30.63 *	1.06 *	70.48%
15–19 years	PE-INAR(1)-X	−0.19	1.51	2.93 *	1.41 *	2.35 *	−0.14	−0.97	−0.62 *	63.52%
	PL-INAR(1)-X	−0.20	1.51	2.88 *	1.41 *	1.74 *	−0.13	−0.85	−0.57 *	**63.52%**
Total	PE-INAR(1)-X	−0.10	1.50	22.46 *	0.18	3.73 *	−0.19	−17.95 *	0.27 *	67.76%
	PL-INAR(1)-X	−0.10	1.50	21.06 *	0.18	3.06 *	−0.19	−15.78 *	0.26 *	**67.76%**

Notes: PE and PL, respectively, stand for the Poisson–exponential and Poisson–Lindley error distributions for the INAR(1)-X model $Y_{t}=\beta_{t}\circ Y_{t-1}+\varepsilon_{t}$, as characterised in Table 1. Meanwhile, NSE is the Nash–Sutcliffe efficiency, as formulated in Equation (A6) in Appendix A; the highest NSE is given in boldface. The parameters $b_{0},b_{1},b_{2},b_{3}$ determine the probability $\beta_{t}=1/[1+\exp\left(-\left(b_{0}+b_{1}X_{1,t-1}+b_{2}X_{2,t-1}+b_{3}X_{3,t-1}\right)\right)]$ that a child will still have diarrhoea from month t−1 to month *t*, where $X_{1,t-1},X_{2,t-1},X_{3,t-1}$, respectively, denote the numbers of floods, landslides, and extreme weather events in the previous month t−1. Meanwhile, $c_{0},c_{1},c_{2},c_{3}$ determine the parameter $\gamma_{t}=\exp\left(-\left(c_{0}+c_{1}X_{1,t-1}+c_{2}X_{2,t-1}+c_{3}X_{3,t-1}\right)\right)$ of the error or additional number $\varepsilon_{t}$ of diarrhoea cases from month t−1 to month *t*. Positive (negative) $b_{j}$ and $c_{j}$ indicate that an increase in $X_{j,t-1}$ results in an increase (decrease) in the probability $\beta_{t}$ and the mean $E(\varepsilon_{t})$. The asterisk * suggests that $b_{j}$ and $c_{j}$ are significantly nonzero at the 5% significance level, implying that there is a significant effect of $X_{j,t-1}$ on $\beta_{t}$ and $E(\varepsilon_{t})$, also on $E(Y_{t})$.

**Table 6:** Estimated parameters and Nash–Sutcliffe efficiency of INAR(1)-X models for the number of ARI cases among children over Period 1 (before the COVID-19 pandemic) and Period 2 (during and after the COVID-19 pandemic).

Age Group	Model	${\hat{b}}_{0}$	${\hat{b}}_{1}$	${\hat{b}}_{2}$	${\hat{b}}_{3}$	${\hat{c}}_{0}$	${\hat{c}}_{1}$	${\hat{c}}_{2}$	${\hat{c}}_{3}$	NSE
Period 1										
0–7 days	PE-INAR(1)-X	0.12	−0.57 *	1.19	0.65	2.70 *	0.18 *	−2.30 *	−0.39	25.16%
	PL-INAR(1)-X	0.12	−0.58 *	1.22	0.66	2.06 *	0.17 *	−2.20 *	−0.37	**25.16%**
8–28 days	PE-INAR(1)-X	0.25	−0.39 *	2.13 *	−0.09	4.58 *	0.17 *	−1.76 *	0.03	**29.68%**
	PL-INAR(1)-X	0.25	−0.39 *	2.13 *	−0.09	3.90 *	0.17 *	−1.73 *	0.03	29.68%
29 days–11 months	PE-INAR(1)-X	1.25 *	1.40 *	0.49 *	−1.66 *	7.03 *	−1.19 *	−1.97 *	1.04 *	66.50%
	PL-INAR(1)-X	1.25 *	1.40 *	0.49 *	−1.66 *	6.34 *	−1.19 *	−1.96 *	1.04 *	**66.51%**
12 months–4 years	PE-INAR(1)-X	1.02 *	1.36 *	0.62 *	−1.62 *	8.10 *	−1.12 *	−3.84	0.95 *	**60.74%**
	PL-INAR(1)-X	4.04 *	−0.20 *	−0.85 *	0.20	−44.08 *	−12.02 *	29.13 *	−6.51 *	50.89%
5–9 years	PE-INAR(1)-X	0.68 *	−0.06	1.71 *	0.40	7.93 *	0.01	−11.12	−0.07	**53.16%**
	PL-INAR(1)-X	0.90 *	−0.18 *	2.18 *	35.07 *	7.11 *	0.08 *	−24.35 *	−3.40	50.92%
10–14 years	PE-INAR(1)-X	0.94 *	−0.14 *	1.64 *	180.97 *	7.21 *	0.05 *	−54.85 *	−2.47	55.06%
	PL-INAR(1)-X	0.79 *	−0.06 *	1.38 *	0.40	6.62 *	−0.02	−16.75	0.02	**57.71%**
15–19 years	PE-INAR(1)-X	0.76 *	−0.18 *	2.25 *	5797.69	7.20 *	0.07 *	−17.44 *	−0.70	44.38%
	PL-INAR(1)-X	0.76 *	−0.18 *	2.25 *	103.77 *	6.50 *	0.07 *	−19.89 *	−0.69	**44.38%**
Total	PE-INAR(1)-X	0.98 *	−0.01	1.32 *	0.32	9.17 *	−0.03	−23.26 *	0.01	61.66%
	PL-INAR(1)-X	1.20 *	3.82 *	0.22	−1.79 *	8.35 *	−39.82 *	−13.84	1.01 *	**63.72%**
Period 2										
0–7 days	PE-INAR(1)-X	−0.07	−0.01	−24.70 *	1.17 *	1.40 *	0.11	−0.06	−0.55	**15.14%**
	PL-INAR(1)-X	−0.62	0.54 *	−1.42 *	0.10	1.16 *	−0.19	−0.53	−0.58 *	14.98%
8–28 days	PE-INAR(1)-X	−0.72	1.94 *	−0.62	−6.82 *	2.92 *	−0.03	−0.01	0.33 *	21.81%
	PL-INAR(1)-X	−0.72	1.94 *	−0.62	−6.82 *	2.27 *	−0.02	−0.01	0.32 *	**21.82%**
29 days–11 months	PE-INAR(1)-X	2.41 *	−0.32	44.49 *	4465.52	5.10 *	0.15	−0.48	0.08	86.86%
	PL-INAR(1)-X	2.41 *	−0.32	42.20 *	373.05 *	4.41 *	0.15	−0.48	0.08	**86.87%**
12 months–4 years	PE-INAR(1)-X	2.51 *	−0.45 *	15.47 *	32.48 *	6.28 *	0.10	−0.29	0.25	88.36%
	PL-INAR(1)-X	2.51 *	−0.45 *	73.46 *	22.66 *	5.59 *	0.10	−0.29	0.25	**88.36%**
5–9 years	PE-INAR(1)-X	1.95 *	0.08	127.58 *	367.45 *	6.80 *	−1.46	−0.36	−16.74 *	85.88%
	PL-INAR(1)-X	2.04 *	0.10	20.72 *	21.11 *	6.07 *	−2.21	−0.26	0.36	**86.65%**
10–14 years	PE-INAR(1)-X	1.58 *	0.40	251.09	3278.54	6.46 *	−4.62	−0.14	−0.45	**83.93%**
	PL-INAR(1)-X	2.98 *	−0.21	127.01 *	290.81	−82.57 *	4.49 *	24.58 *	2.62 *	81.53%
15–19 years	PE-INAR(1)-X	1.98 *	−0.40 *	19.52 *	63.28 *	5.68 *	0.04	−0.21	−0.01	85.20%
	PL-INAR(1)-X	1.98 *	−0.40 *	33.84 *	60.48 *	4.99 *	0.04	−0.21	−0.02	**85.20%**
Total	PE-INAR(1)-X	2.13 *	0.01	15.20 *	52.47 *	7.96 *	−1.49	−0.26	0.11	**88.14%**
	PL-INAR(1)-X	3.58 *	−0.51 *	18.71 *	90.44 *	−9.81 *	−1.86	−4.04 *	−14.85 *	86.59%

Notes: PE and PL, respectively, stand for the Poisson–exponential and Poisson–Lindley error distributions for the INAR(1)-X model $Y_{t}=\beta_{t}\circ Y_{t-1}+\varepsilon_{t}$, as characterised in Table 1. Meanwhile, NSE is the Nash–Sutcliffe efficiency, as formulated in Equation (A6) in Appendix A; the highest NSE is given in boldface. The parameters $b_{0},b_{1},b_{2},b_{3}$ determine the probability $\beta_{t}=1/[1+\exp\left(-\left(b_{0}+b_{1}X_{1,t-1}+b_{2}X_{2,t-1}+b_{3}X_{3,t-1}\right)\right)]$ that a child will still have diarrhoea from month t−1 to month *t*, where $X_{1,t-1},X_{2,t-1},X_{3,t-1}$, respectively, denote the numbers of floods, landslides, and extreme weather events in the previous month t−1. Meanwhile, $c_{0},c_{1},c_{2},c_{3}$ determine the parameter $\gamma_{t}=\exp\left(-\left(c_{0}+c_{1}X_{1,t-1}+c_{2}X_{2,t-1}+c_{3}X_{3,t-1}\right)\right)$ of the error or additional number $\varepsilon_{t}$ of diarrhoea cases from month t−1 to month *t*. Positive (negative) $b_{j}$ and $c_{j}$ indicate that an increase in $X_{j,t-1}$ results in an increase (decrease) in the probability $\beta_{t}$ and the mean $E(\varepsilon_{t})$. The asterisk * suggests that $b_{j}$ and $c_{j}$ are significantly nonzero at the 5% significance level, implying that there is a significant effect of $X_{j,t-1}$ on $\beta_{t}$ and $E(\varepsilon_{t})$, also on $E(Y_{t})$.

## Data Availability

The data on infectious disease cases among children analysed in this study were retrieved from the epidemiological surveillance and immunisation information system database, particularly the healthcare centres’ integrated disease surveillance database, maintained by the Health Office of the Province of the Special Region of Jakarta, Indonesia (https://surveilans-dinkes.jakarta.go.id, accessed on 17 March 2025). Meanwhile, the disaster data for this province were collected from the Indonesia Disaster Information Database managed by the National Disaster Management Agency (BNPB) of the Republic of Indonesia (https://dibi.bnpb.go.id, accessed on 17 March 2025).

## Supplementary Materials

The following supporting information can be downloaded at https://www.mdpi.com/article/10.3390/diseases13090303/s1.

## Abbreviations

The following abbreviations are used in this manuscript:

ARI	Acute respiratory infection
DHF	Dengue haemorrhagic fever
INAR	Integer-valued autoregressive
INAR-X	Integer-valued autoregressive with explanatory variables
NSE	Nash–Sutcliffe efficiency
PE	Poisson–exponential
PL	Poisson–Lindley

## Appendix A. Estimation for INAR-X Models

Let {*Y*_1_, *Y*_2_,…, *Y_n_*} denote a sample of size *n* with *Y_t_* denoting the actual number of infectious disease cases among children at time *t*. Suppose that ${\hat{Y}}_{t}$ denotes its fitted value according to an INAR(1)-X model, that is, ${\hat{Y}}_{t}=E\left(Y_{t}|Y_{t-1}\right)=\beta_{t}Y_{t-1}+E\left(\varepsilon_{t}\right)$. The corresponding error is thus given by

(A1) $$e_{t}=Y_{t}-{\hat{Y}}_{t}=Y_{t}-\beta_{t}Y_{t-1}-E\left(\varepsilon_{t}\right).$$

Using the conditional least-squares estimation method, we can determine an estimator $\hat{\theta }={\left({\hat{b}}_{0},{\hat{b}}_{1},\ldots ,{\hat{b}}_{m},{\hat{c}}_{0},{\hat{c}}_{1},\ldots ,{\hat{c}}_{m}\right)}^{\prime }$ for the parameter vector $\theta ={\left(b_{0},b_{1},\ldots ,b_{m},c_{0},c_{1},\ldots ,c_{m}\right)}^{\prime }\in R^{2m+2}$ of the INAR(1)-X model by minimising the following sum of squared errors: $\sum_{t=2}^{n}e_{t}^{2}$. This means that it can be derived by finding a solution to

(A2) $$\sum_{t=2}^{n}\frac{\partial e_{t}^{2}}{\partial \theta }=0,$$

evaluated at $\theta =\hat{\theta }$. It is worth mentioning that its asymptotic normality is given by $\sqrt{n-1}\left(\hat{\theta }-\theta \right)\overset{d}{\to }N_{2m+2}\left(0,\sum \right)$ as *n* approaches infinity. By adopting *M*-estimation theory in van der Vaart [72], we can estimate the covariance matrix **∑** by $\hat{\sum }={\hat{V}}^{-1}\hat{U}{\left({\hat{V}}^{\prime }\right)}^{-1}$, with

(A3) $$\begin{array}{cc} U & =\frac{1}{n-1}\sum_{t=2}^{n}\frac{\partial e_{t}^{2}}{\partial \theta }\;\frac{\partial e_{t}^{2}}{\partial \theta^{\prime }}=\frac{4}{n-1}\sum_{t=2}^{n}e_{t}^{2}\;\frac{\partial e_{t}}{\partial \theta }\;\frac{\partial e_{t}}{\partial \theta^{\prime }}, \end{array}$$

(A4) $$\begin{array}{cc} V & =\frac{1}{n-1}\sum_{t=2}^{n}\frac{\partial^{2}e_{t}^{2}}{\partial \theta^{2}}=\frac{2}{n-1}\sum_{t=2}^{n}\left({\left(\frac{\partial e_{t}}{\partial \theta }\right)}^{2}+e_{t}\;\frac{\partial^{2}e_{t}}{\partial \theta^{2}}\right). \end{array}$$

In particular, for some $\theta_{j}=b_{j}$ or $\theta_{j}=c_{j}$, we have $\sqrt{n-1}\left({\hat{\theta }}_{j}-\theta_{j}\right)\overset{d}{\to }N\left(0,\sigma_{j}^{2}\right)$, with

(A5) $$\sigma_{j}^{2}=\frac{\left(n-1\right)\sum_{t=2}^{n}e_{t}^{2}{\left(\frac{\partial e_{t}}{\partial \theta_{j}}\right)}^{2}}{{\left[\sum_{t=2}^{n}\left({\left(\frac{\partial e_{t}}{\partial \theta_{j}}\right)}^{2}+e_{t}\;\frac{\partial^{2}e_{t}}{\partial \theta_{j}^{2}}\right)\right]}^{2}}.$$

Accordingly, we can examine the null hypothesis H_0_: *θ_j_* = 0 using a *t*-test with test statistic $T=\sqrt{n-1}\;{\hat{\theta }}_{j}/{\hat{\sigma }}_{j}$, which asymptotically follows a *t*-distribution with n−(2m+2)−1 degrees of freedom. To compare some INAR(1)-X models under consideration, we can compare their Nash–Sutcliffe efficiency (NSE) defined as

(A6) $$\mathrm{NSE}=1-\frac{\sum_{t=2}^{n}{\left(Y_{t}-{\hat{Y}}_{t}\right)}^{2}}{\sum_{t=2}^{n}{\left(Y_{t}-\bar{Y}\right)}^{2}},$$

where $\bar{Y}$ denotes the sample mean of {*Y*_1_, *Y*_2_,…, *Y_n_*}.
