# Supplementary material for: Infectious Diseases in Children: Diagnosing the Impact of Climate Change-Related Disasters Using Integer-Valued Autoregressive Models with Overdispersion
Source: Diseases. 2025 Sep 15;13(9):303. doi: 10.3390/diseases13090303 (PMC12468014; doi:10.3390/diseases13090303)
Supplement: Supplementary file 1 [file diseases-13-00303-s001.zip › diseases-3787654-supplementary.pdf]

Article

Infectious Diseases in Children: Diagnosing the Impact of Climate Change-Related Disasters Using Integer-Valued Autoregressive Models with Overdispersion

Dessie Wanda [1], Holivia Almira Jacinta [1], Arief Rahman Hakim [2], Atina Ahdika [3], Suryane Sulistiana Susanti [1,*] and Khreshna Syuhada [4]

[1] Faculty of Nursing, Universitas Indonesia, Depok, 16424, Indonesia; dessie@ui.ac.id (D.W.); holivia.almira@ui.ac.id (H.A.J.)
[2] Research Centre for Computing, National Research and Innovation Agency (BRIN), Bandung, 40135, Indonesia; arief.rahman.hakim@brin.go.id
[3] Department of Statistics, Universitas Islam Indonesia, Yogyakarta, 55584, Indonesia; atina.a@uii.ac.id
[4] Statistics Research Division, Institut Teknologi Bandung, Bandung, 40132, Indonesia; khreshna@itb.ac.id
* Correspondence: suryane.s.susanti@ui.ac.id

Supplementary Materials

S.1 Infectious Disease and Disaster Distributions Among Children in Jakarta in 2024

Received:
Revised:
Accepted:
Published:

Citation: Wanda, D.; Jacinta, H. A.; Hakim, A. R.; Ahdika, A.; Susanti, S. S.; Syuhada, K. Infectious Diseases in Children: Diagnosing the Impact of Climate Change-Related Disasters Using Integer-Valued Autoregressive Models with Overdispersion. *Diseases* **2025**, *1*, 0. https://doi.org/

Copyright: © 2025 by the authors. Submitted to *Diseases* for possible open access publication under the terms and conditions of the Creative Commons Attri- bution (CC BY) license (https://creativecommons.org/licenses/by/4.0/).

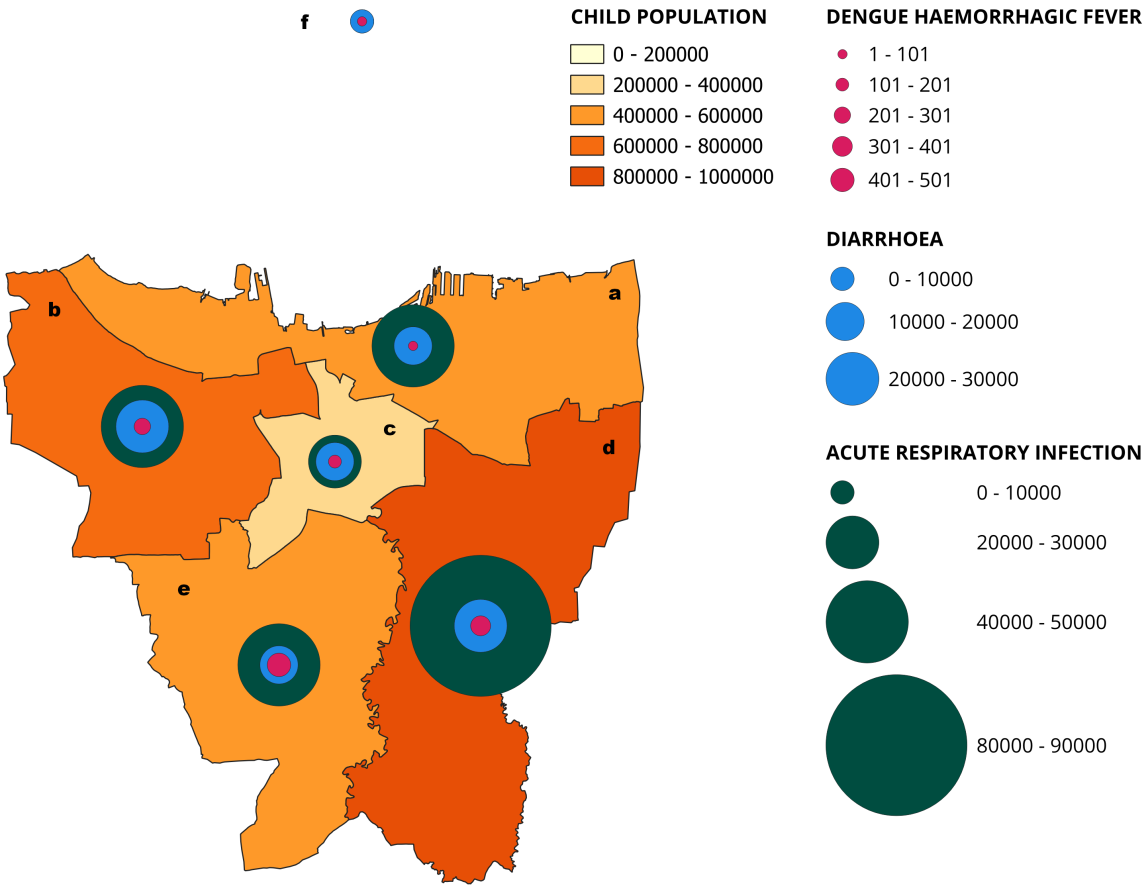

Figure S1. Comparative ratio between the incidences of three infectious diseases and the child population in Jakarta in 2024. *Notes*: Letters on the map indicate the cities of Jakarta: (a) North Jakarta, (b) West Jakarta, (c) Central Jakarta, (d) East Jakarta, (e) South Jakarta, and (f) Seribu Islands Administrative Regency.

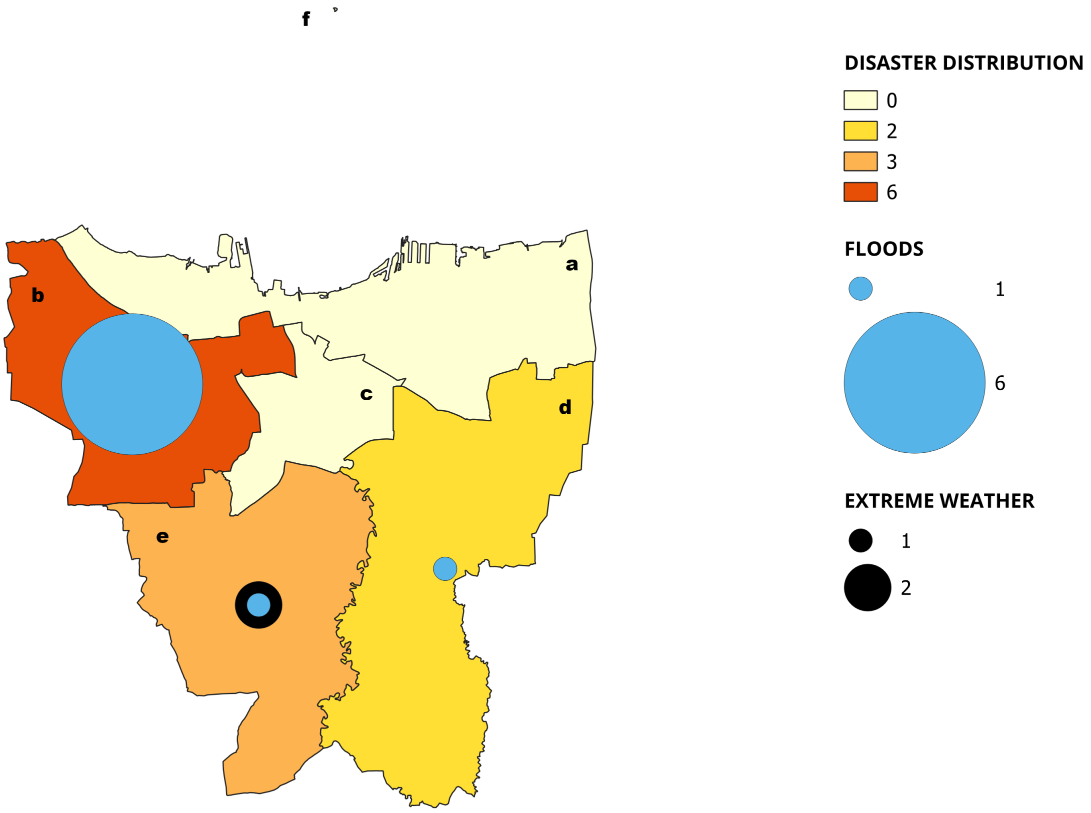

Figure S2. Comparison between disaster occurrences in Jakarta in 2024. *Notes*: The figure displays only two types of disasters, as the DIBI database indicates that no landslide events were recorded in Jakarta during that year. Letters on the map indicate the cities of Jakarta: (a) North Jakarta, (b) West Jakarta, (c) Central Jakarta, (d) East Jakarta, (e) South Jakarta, and (f) Seribu Islands Administrative Regency.

S.2 Autocorrelation and Partial Autocorrelation Functions of Disease and Disaster Data

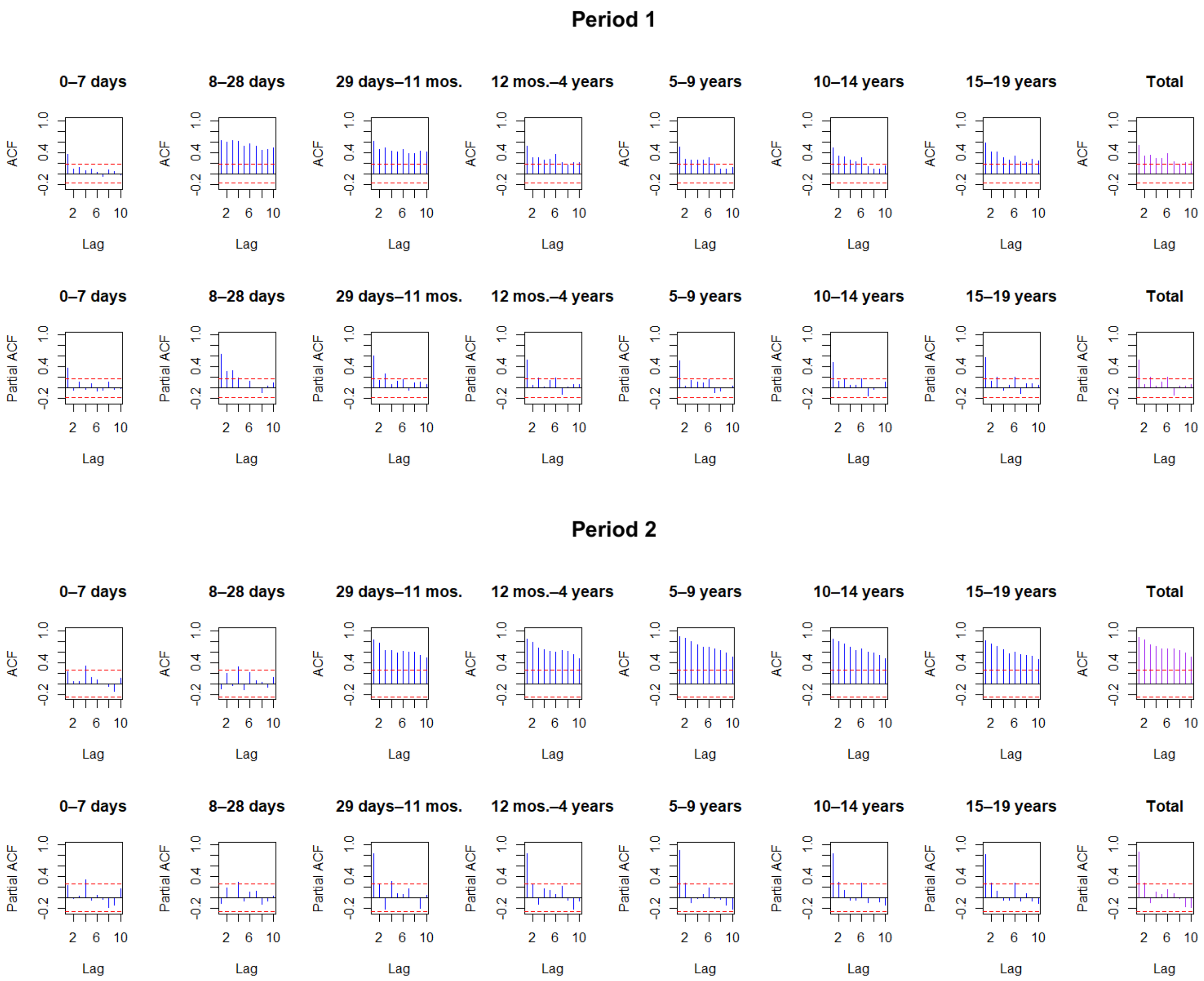

Figure S3. Autocorrelation function (ACF) and partial ACF of count time-series data on diarrhoea cases among children over Period 1 (before the COVID-19 pandemic) and Period 2 (during and after the COVID-19 pandemic). *Notes*: The dashed red lines are their 95% lower and upper confidence limits.

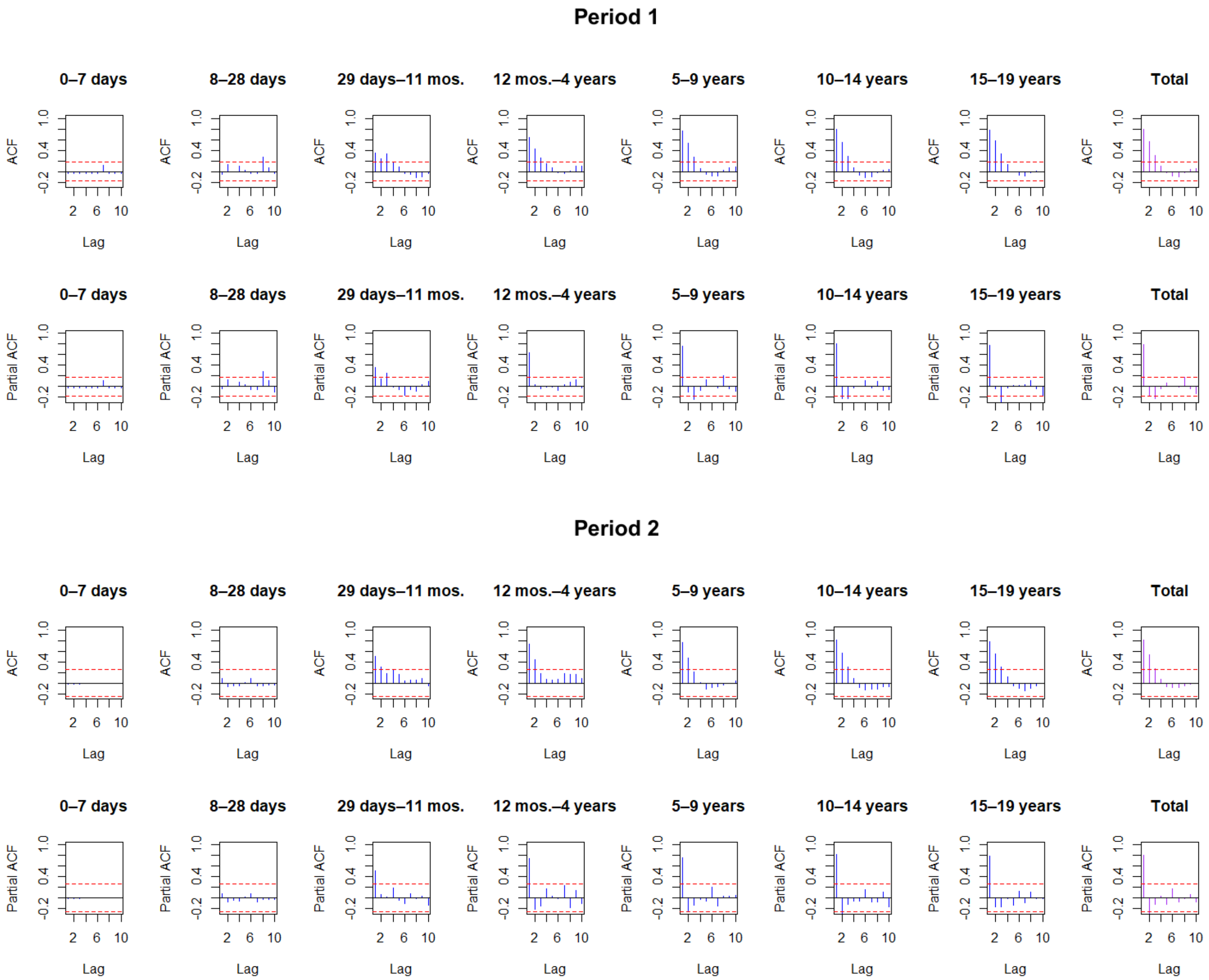

Figure S4. Autocorrelation function (ACF) and partial ACF of count time-series data on DHF cases among children over Period 1 (before the COVID-19 pandemic) and Period 2 (during and after the COVID-19 pandemic). *Notes*: The dashed red lines are their 95% lower and upper confidence limits.

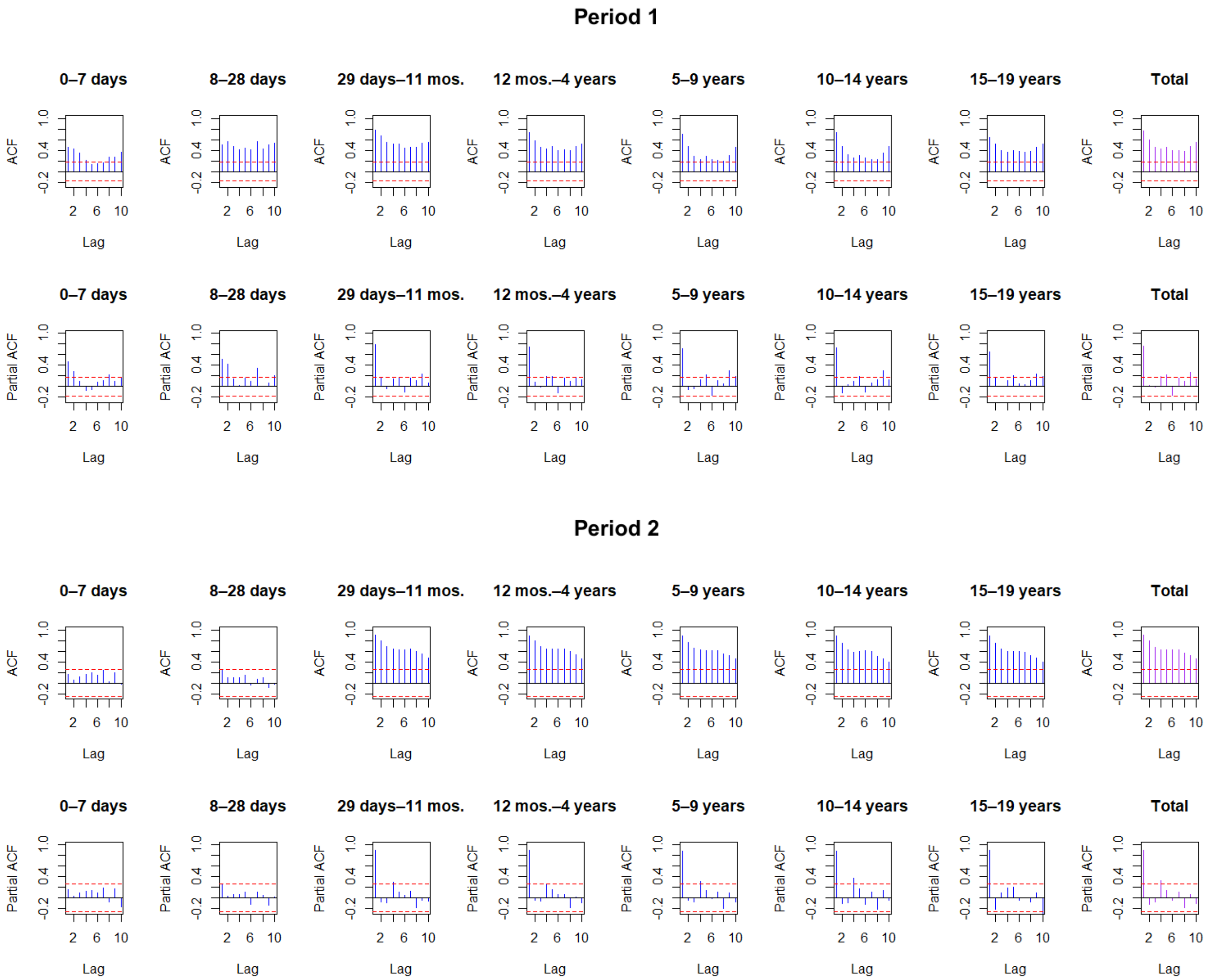

Figure S5. Autocorrelation function (ACF) and partial ACF of count time-series data on ARI cases among children over Period 1 (before the COVID-19 pandemic) and Period 2 (during and after the COVID-19 pandemic). *Notes*: The dashed red lines are their 95% lower and upper confidence limits.

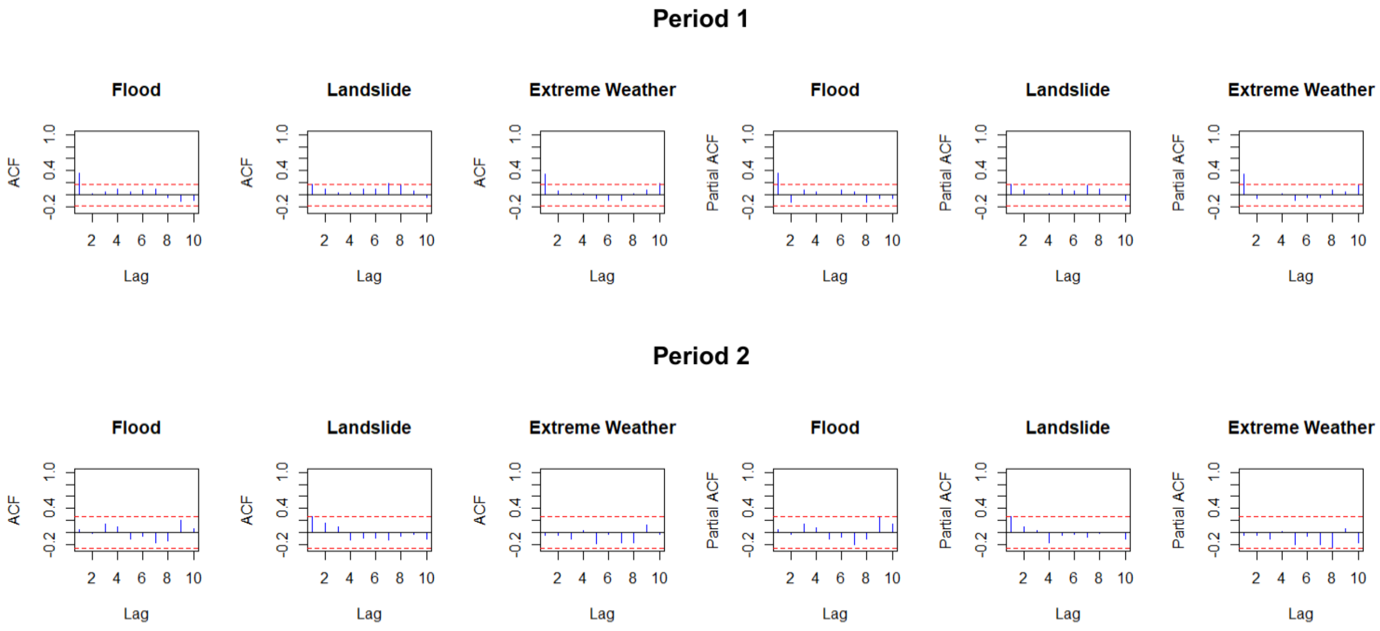

Figure S6. Autocorrelation function (ACF) and partial ACF of count time-series data on natural disasters over Period 1 (before the COVID-19 pandemic) and Period 2 (during and after the COVID-19 pandemic). *Notes*: The dashed red lines are their 95% lower and upper confidence limits.

S.3 Cross-Correlation Functions Between Disease Data and Disaster Data

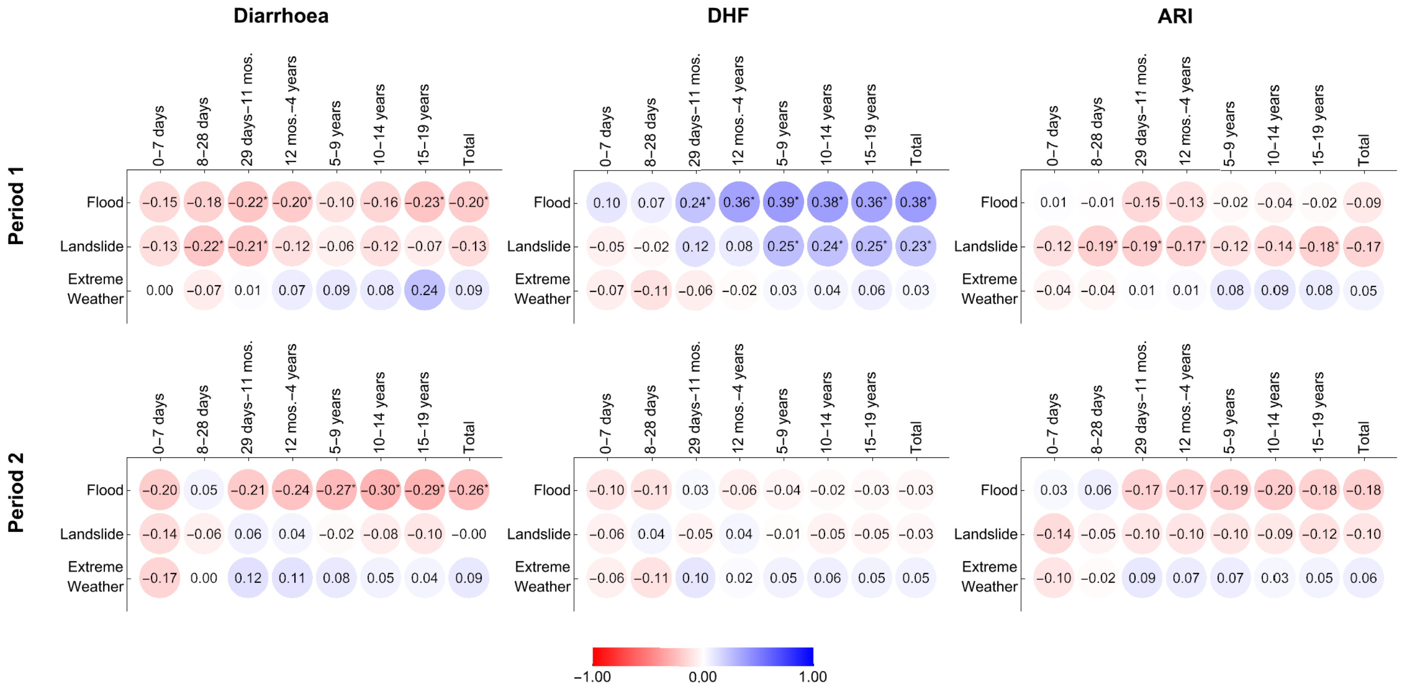

Figure S7. Cross-correlation function between (i) the number of infectious disease cases in children in month t and (ii) the number of natural disasters in the previous month $t-1$ over Period 1 (before the COVID-19 pandemic) and Period 2 (during and after the COVID-19 pandemic). *Notes*: The asterisk * indicates that the cross-correlation function is significantly nonzero at the 5% significance level.

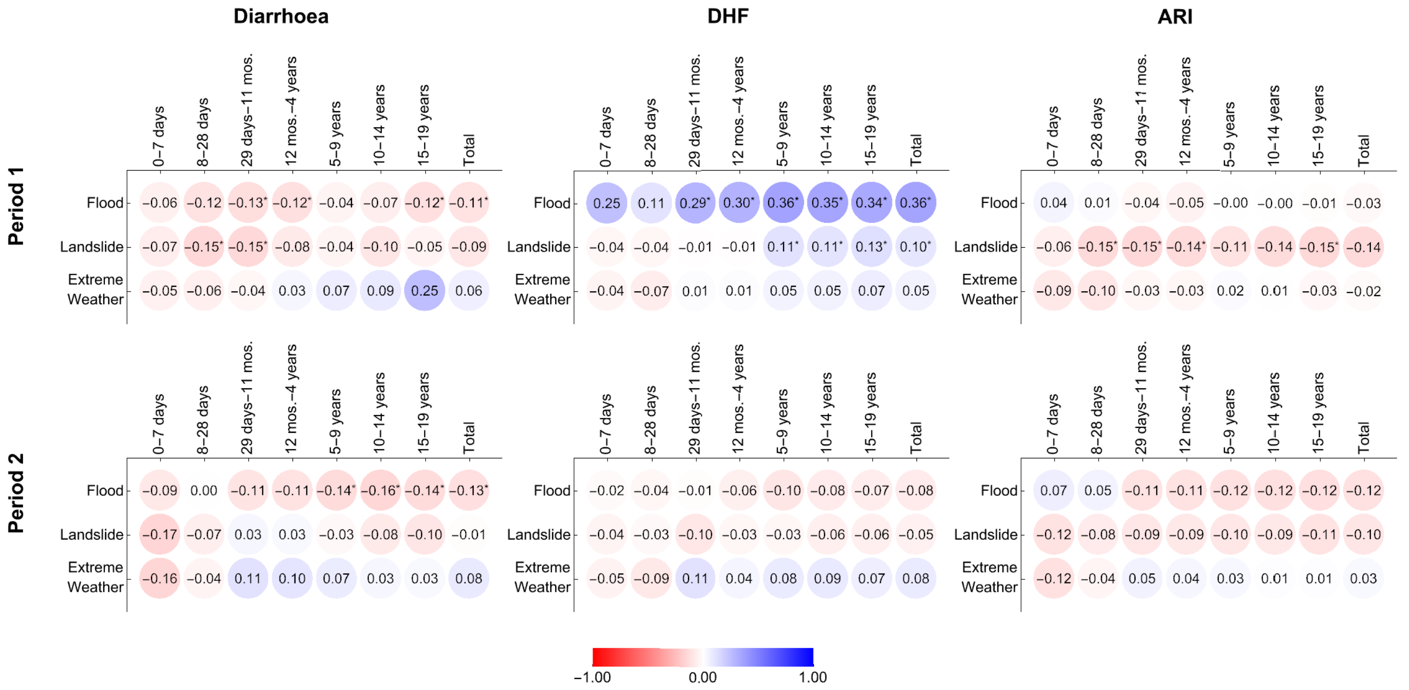

Figure S8. Cross-correlation function between (i) the number of infectious disease cases in children in month t and (ii) the exponential transformation of the number of natural disasters in the previous month $t-1$ over Period 1 (before the COVID-19 pandemic) and Period 2 (during and after the COVID-19 pandemic). *Notes*: The asterisk * indicates that the cross-correlation function is significantly nonzero at the 5% significance level.

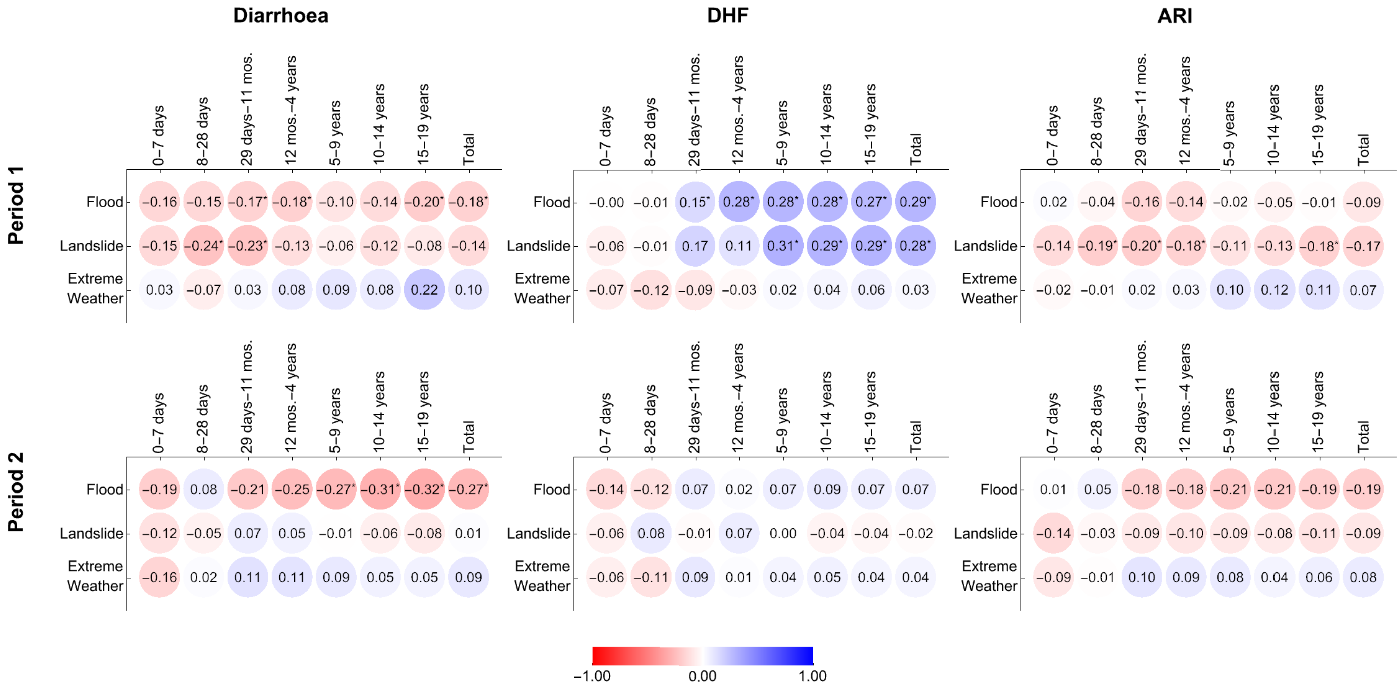

Figure S9. Cross-correlation function between (i) the number of infectious disease cases in children in month t and (ii) the logistic transformation of the number of natural disasters in the previous month $t - 1$ over Period 1 (before the COVID-19 pandemic) and Period 2 (during and after the COVID-19 pandemic). *Notes*: The asterisk * indicates that the cross-correlation function is significantly nonzero at the 5% significance level.

S.4 Autocorrelation and Partial Autocorrelation Functions of Error Data 5

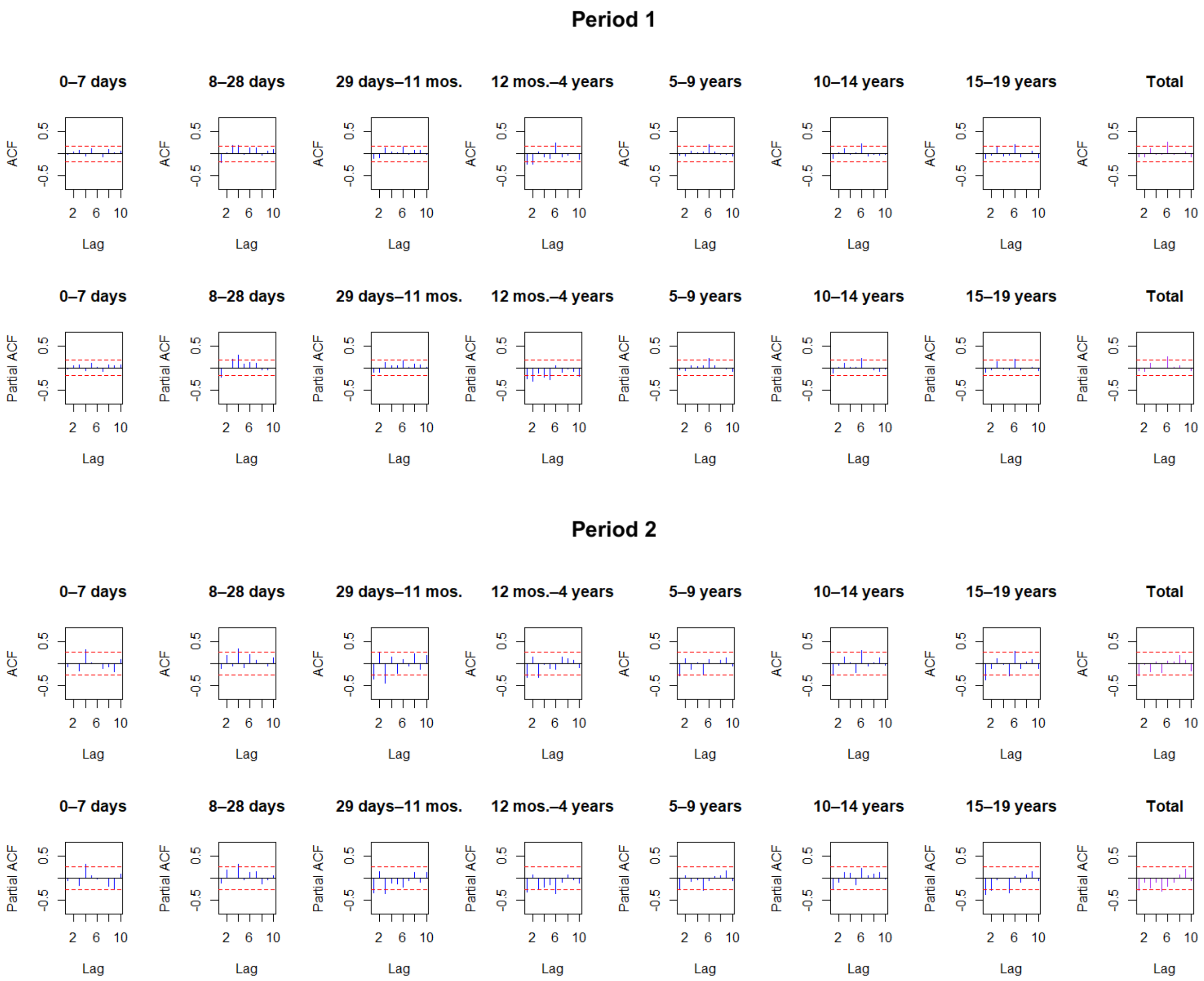

Figure S10. Autocorrelation function (ACF) and partial ACF of error data resulting from a PE-INAR(1)-X model for the number of diarrhoea cases among children over Period 1 (before the COVID-19 pandemic) and Period 2 (during and after the COVID-19 pandemic). *Notes*: The dashed red lines are their 95% lower and upper confidence limits.

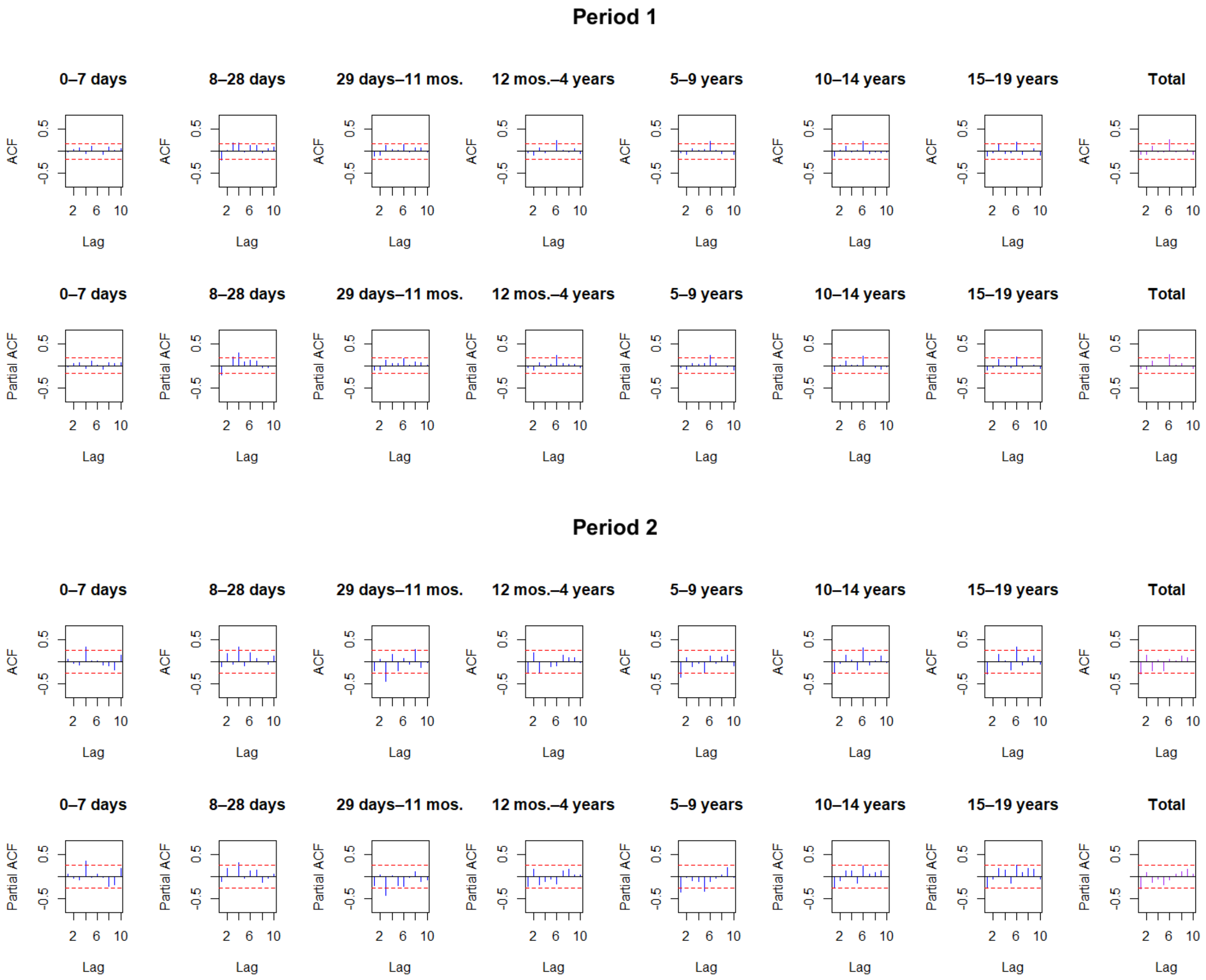

Figure S11. Autocorrelation function (ACF) and partial ACF of error data resulting from a PL-INAR(1)-X model for the number of diarrhoea cases among children over Period 1 (before the COVID-19 pandemic) and Period 2 (during and after the COVID-19 pandemic). *Notes*: The dashed red lines are their 95% lower and upper confidence limits.

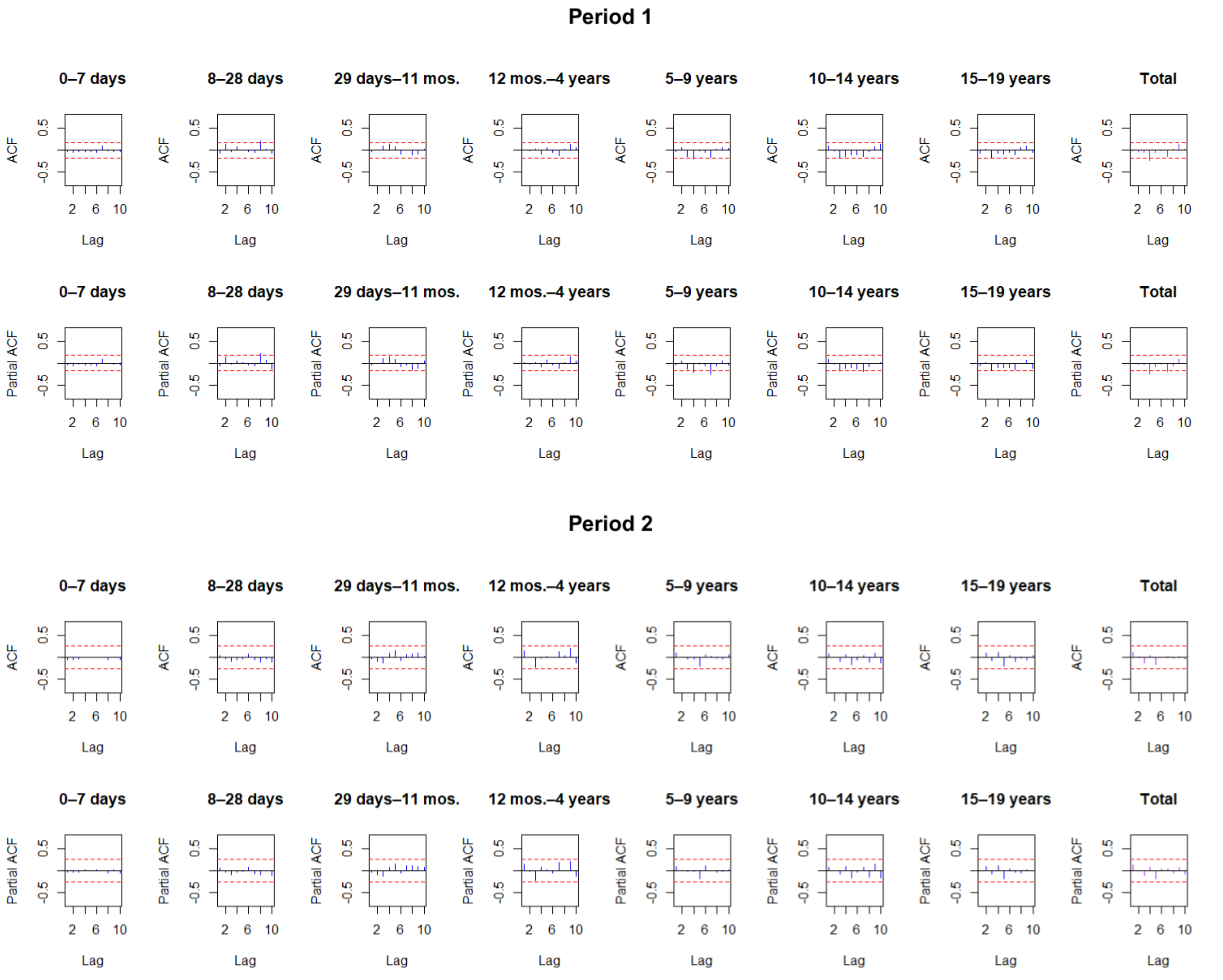

Figure S12. Autocorrelation function (ACF) and partial ACF of error data resulting from a PE-INAR(1)-X model for the number of DHF cases among children over Period 1 (before the COVID-19 pandemic) and Period 2 (during and after the COVID-19 pandemic). *Notes*: The dashed red lines are their 95% lower and upper confidence limits.

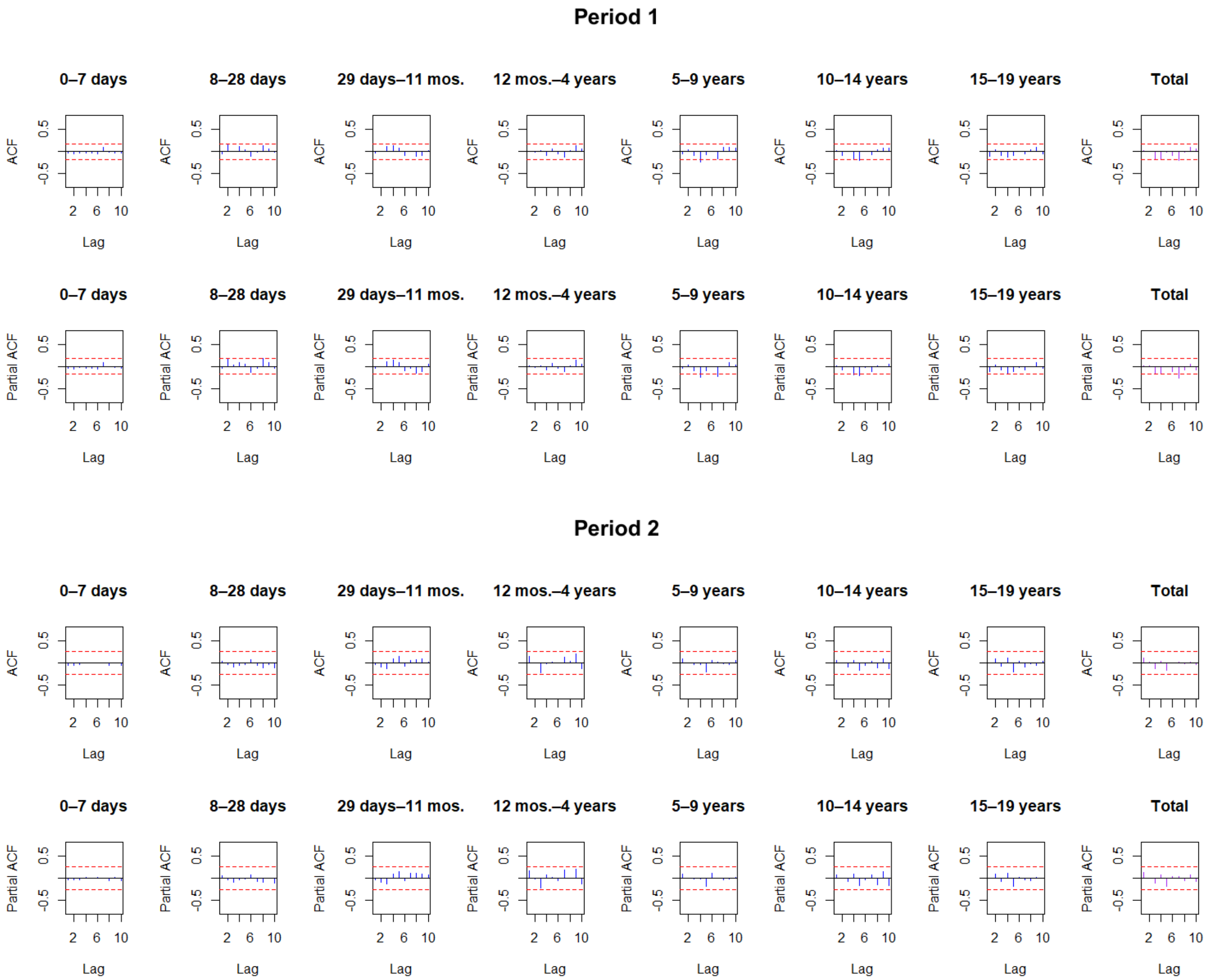

Figure S13. Autocorrelation function (ACF) and partial ACF of error data resulting from a PL-INAR(1)-X model for the number of DHF cases among children over Period 1 (before the COVID-19 pandemic) and Period 2 (during and after the COVID-19 pandemic). *Notes*: The dashed red lines are their 95% lower and upper confidence limits.

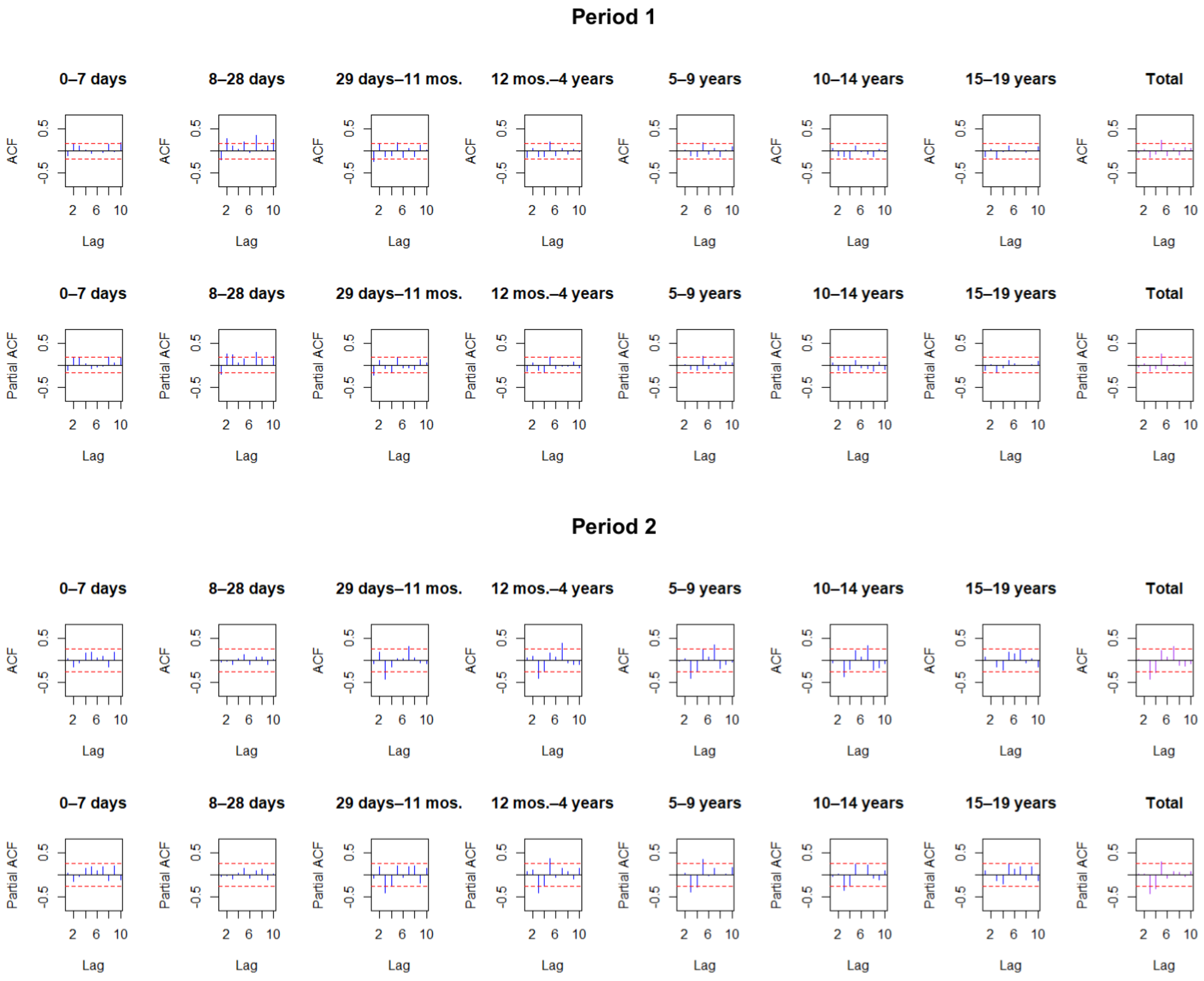

Figure S14. Autocorrelation function (ACF) and partial ACF of error data resulting from a PE-INAR(1)-X model for the number of ARI cases among children over Period 1 (before the COVID-19 pandemic) and Period 2 (during and after the COVID-19 pandemic). *Notes*: The dashed red lines are their 95% lower and upper confidence limits.

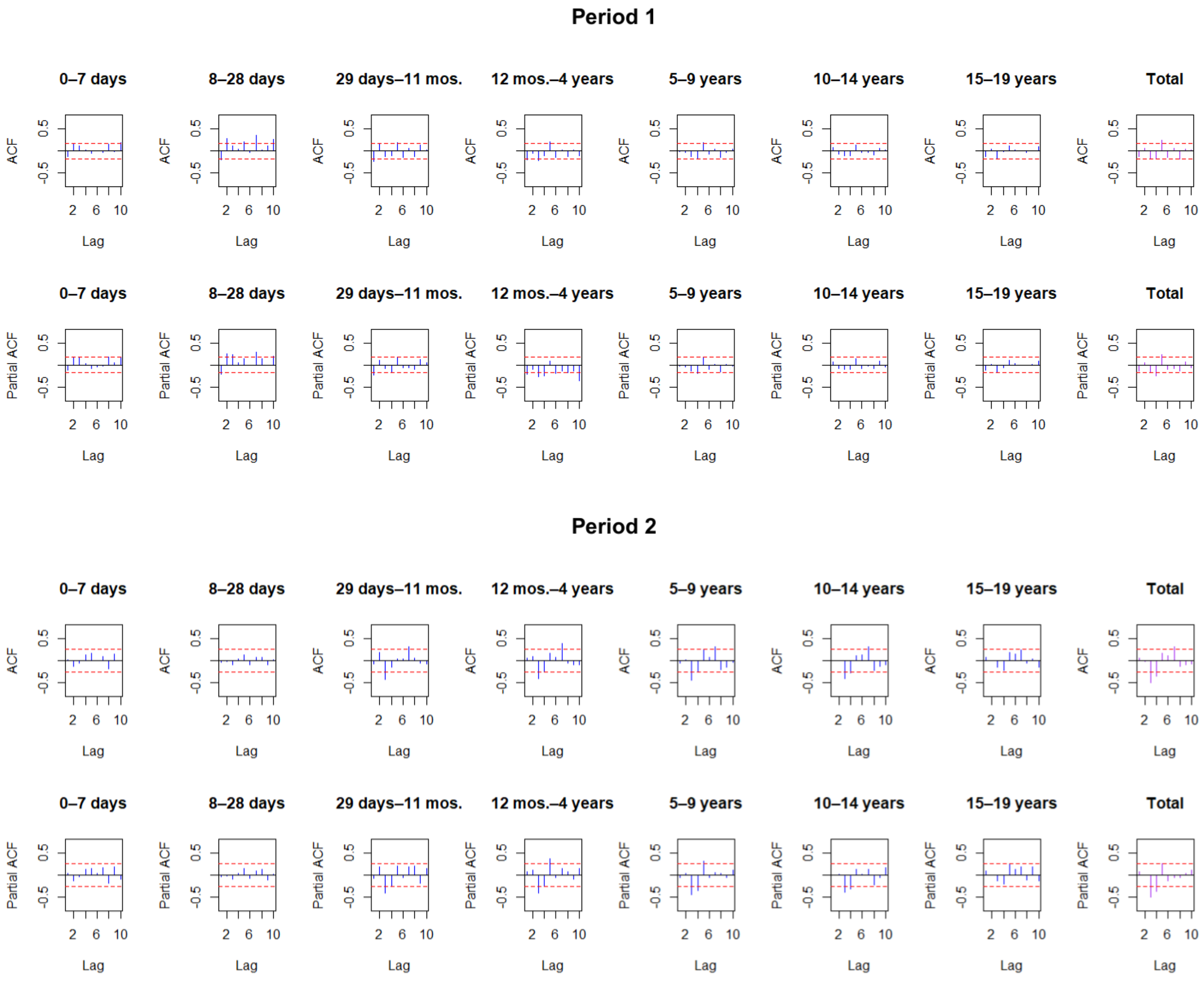

Figure S15. Autocorrelation function (ACF) and partial ACF of error data resulting from a PL-INAR(1)-X model for the number of ARI cases among children over Period 1 (before the COVID-19 pandemic) and Period 2 (during and after the COVID-19 pandemic). *Notes*: The dashed red lines are their 95% lower and upper confidence limits.
